# Trade-off between local transmission and long-range dispersal drives infectious disease outbreak size in spatially structured populations

**DOI:** 10.1371/journal.pcbi.1008009

**Published:** 2020-07-06

**Authors:** Elisa Benincà, Thomas Hagenaars, Gert Jan Boender, Jan van de Kassteele, Michiel van Boven

**Affiliations:** 1 Centre for Infectious Disease Control, National Institute for Public Health and the Environment, The Netherlands; 2 Department of Bacteriology and Epidemiology, Wageningen Bioveterinary Research, Lelystad, The Netherlands; The Pennsylvania State University, UNITED STATES

## Abstract

Transmission of infectious diseases between immobile hosts (e.g., plants, farms) is strongly dependent on the spatial distribution of hosts and the distance-dependent probability of transmission. As the interplay between these factors is poorly understood, we use spatial process and transmission modelling to investigate how epidemic size is shaped by host clustering and spatial range of transmission. We find that for a given degree of clustering and individual-level infectivity, the probability that an epidemic occurs after an introduction is generally higher if transmission is predominantly local. However, local transmission also impedes transfer of the infection to new clusters. A consequence is that the total number of infections is maximal if the range of transmission is intermediate. In highly clustered populations, the infection dynamics is strongly determined by the probability of transmission between clusters of hosts, whereby local clusters act as multiplier of infection. We show that in such populations, a metapopulation model sometimes provides a good approximation of the total epidemic size, using probabilities of local extinction, the final size of infections in local clusters, and probabilities of cluster-to-cluster transmission. As a real-world example we analyse the case of avian influenza transmission between poultry farms in the Netherlands.

## Author summary

Transmission of infectious diseases between immobile hosts depends on the transmission characteristics of the infection and on the spatial distribution of hosts. Examples include infectious diseases of plants that are spread by wind or via vectors (e.g., Asiatic citrus canker spread between citrus trees), diseases that are transmitted between local host populations (e.g., sylvatic plague transmitted between rodents living in burrows), diseases of production animals that are spread between farms (e.g., avian influenza in poultry transmitted from farm to farm). We use spatial transmission modelling to investigate how the total number of infections over the course of an epidemic is determined by host clustering and spatial range of transmission. We find that for a given degree of clustering and infectivity of hosts, the number of infections is maximal if the spatial range of transmission is intermediate. In highly clustered populations we show that epidemic size can be approximated by a metapopulation model, illustrating that in such populations the transmission dynamics is dominated by transmission between clusters of hosts.

## Introduction

The process of transmission of an infection from one host to the next is central in the epidemiology of infectious diseases. For an infectious disease of hosts that can move around the contact process is a critical factor. For infections that are transmitted between sessile hosts, or populations of hosts at a fixed location, the distance between hosts is often the main factor affecting transmission. Examples include infectious diseases of plants that are spread by wind or via vectors (e.g., Asiatic citrus canker, [1, 2]), diseases that are transmitted between local host populations (e.g., sylvatic plague in feral dogs and gerbils [3, 4]), diseases of production animals that are spread between farms (e.g., avian influenza in poultry;[5, 6]), and transmission of human pathogens between population centres (e.g., measles in the US; [7–10]).

Even though transmission dynamics in populations with immobile hosts is less complex than in populations with mobile hosts, still only a partial understanding exists, and most theoretical analyses make simplifying assumptions from the outset. For instance, the distribution of hosts in space is often not explicitly modelled; instead hosts are classified into one of several subpopulations [11, 12]. In these models, the spatial component is implicitly modelled by determining the connectivity of subpopulations. An alternative in which space is also implicit are the so-called patch-occupancy models. Here, the population is made up of a number of predefined suitable patches that may or may not be occupied by hosts or pathogens [13–17]. There are also models that evaluate the infection dynamics in an explicit spatial setting [5, 18–21]. These models show how local density of hosts and spatial transmission range together determine individual reproduction numbers and areas that are at risk of epidemic transmission. Ultimately, an improved understanding of the factors promoting epidemic transmission can provide an improved basis for the design of effective intervention strategies. Examples where such improvements have been suggested or have even been implemented include diseases of humans, crop and livestock [20–25]. Further, it is now well-recognised that the evolutionary trajectories of pathogens are also moulded by the spatial structure of host populations, thereby, in turn shaping the epidemiological dynamics [26–30].

Inspired by the example of avian influenza transmitted between poultry farms in the Netherlands, we analyse the interplay of host clustering and transmission range on the distribution of outbreak size (i.e. number of hosts that are ultimately infected). For maximal transparency of the arguments, we consider models that include only the essentials of host clustering and that keep the total infection output per infected host constant across transmission range scenarios. Specifically, we study scenarios where the clustering of hosts is described by two parameters, viz. the spatial variance of host density and the spatial range (or scale) over which changes in host density occur. Even though such models do not capture the full complexity of real-world systems, they have the advantage that parameters can be estimated from and compared against real-world data [31], providing an invaluable link between data and model analyses. With regard to the transmission range, we focus on scenarios in which the distance over which transmission occurs varies from highly localised to more dispersed, while keeping the total infectious output per infected host constant. This enables us to study the influence of the range of transmission per se, without complicating the interpretation by simultaneously increasing or decreasing overall transmissibility. Such modelling assumptions are also biologically relevant when pathogen is released from the host in given amounts while being dispersed across a transmission range that is controlled by factors external to the pathogen, e.g., in case of the citrus canker pathogen being transmitted from tree to tree by vectors [32], or avian influenza virus being transmitted from farm to farm by wind [33].

Our analyses uncover a trade-off between local pathogen propagation (which increases with increased local transmission) and infection of novel clusters of hosts (which decreases with increased local transmission) [19, 34]. In highly clustered populations, we show that the size of an epidemic can be approximated using a metapopulation model [35] in which areas with high density of hosts are the main centers of pathogens multiplication, and the surrounding areas with low density of hosts are epidemiologically inert, as they do not support continued pathogen transmission. Finally, we apply our computational approach to the real-world example of avian influenza transmission between poultry farms in the Netherlands, in order to elucidate how in this case transmission is shaped by the highly clustered nature of the Dutch poultry farm population.

### Modelling the spatial distribution of hosts

**Fig 1:**
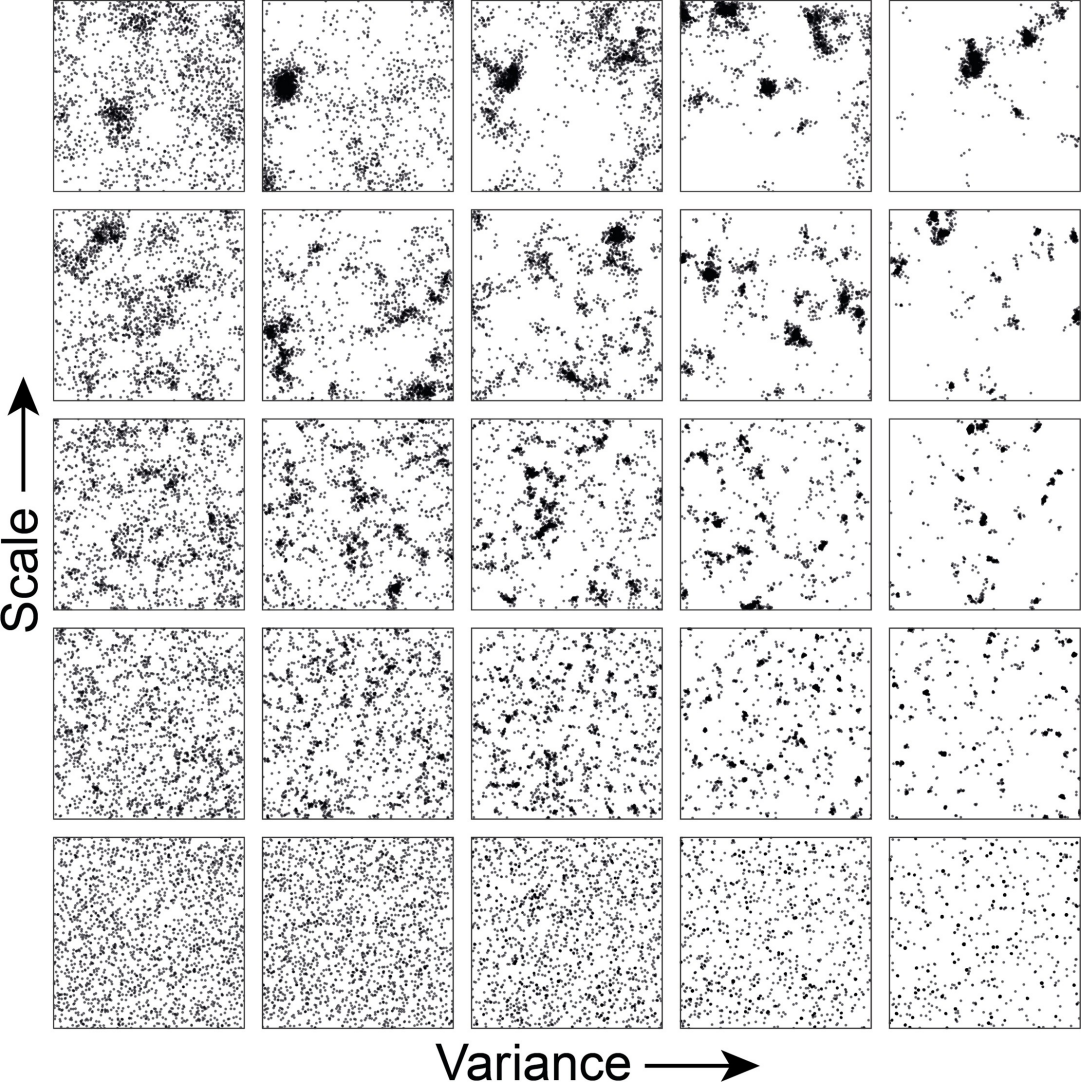
Spatial point patterns of 2,000 hosts in a square of 201 x 201 km. Different clusters patterns have been generated by varying the scale and the variance parameters of the intensity of the random field. The scale parameters are: 0.1; 2; 4; 8; 16 and the variance parameters are 1; 2; 4; 8; 16. By varying the scale and variance of the random field a wide variety of patterns are produced ranging from very homogenous patterns (bottom left hand side) to patterns characterized by huge isolated clusters (top right hand side).

We use a framework that captures a range of possible spatial point patterns, from homogenous to highly clustered, in a systematic way. Specifically, we generate 25 spatial patterns with identical numbers of hosts (*n* = 2,000) and differing only in the level of clustering (Fig 1). Each pattern has been generated by a Log Gaussian Cox process, with an underlying isotropic spatial stochastic process, a random field characterized by a mean log-intensity and a covariance function. Specifically, we generate realizations of random fields on a square grid of 201 km x 201 km by using a covariance model of the Whittle-Matern family. The spatial covariance of this process is described by the function

$$C(r)=\frac{\sigma^{2}}{2^{\left(1-\nu \right)}\Gamma (\nu )}{\left(s\|r\|\right)}^{v}K_{v}(s\|r\|),$$ (1)

where ||*r||* is the distance between two points, Γ and *K*_*v*_ are the Gamma and modified Bessel function of second kind, *ν* is the smoothing parameter, *s* is the scale parameter, and *σ*^2^ is the variance of the random field. Throughout, we take *ν* = 1 and vary the scale and variance parameters. For the scale and variance parameters we take *σ*^2^∈{1,2,4,8,16} and *s*∈ {0.1,2,4,8,16} (Fig A in S1 Supporting Information). The mean intensity of the exponentiated random field is fixed and is equal to $\frac{n_{p}}{n_{g}∙\varepsilon }$ with the number of points *n*_*p*_ = 2,000, the number of grid cells *n*_*g*_ = 40401 cells, and the smearing factor $\varepsilon =e^{\frac{1}{2}\sigma^{2}}$.

We generate different realisations of random field intensity grids with different levels of clustering as a function of two parameters only (Fig B in S1 Supporting Information). Given the intensity in each grid cell, we could sample from a Poisson distribution to generate a point pattern with approximately 2,000 points. However, we exactly require 2,000 points. Therefore, we sample from a Multinomial distribution with size 2,000 and with probabilities equal to the scaled intensities that sum up to one.

### The spatial transmission model

We model the transmission between hosts using a spatially explicit SIR model. At any time *t*, each host is classified in one of the three following states: susceptible (*S*), infected (*I*) or removed (*R*). An uninfected host *j* (state *S*) will be infected by an infected host *i* (state *I*) with a probability *p*(*r*_*ij*_). The probability *p*(*r*_*ij*_) depends on the (Euclidean) distance *r*_*ij*_ = |*r*_*i*_*-r*_*j*_| between the two hosts and on the infectious period *T*_*i*_ of host *i* and is given by:

$$p(r_{ij})=1-e^{-h(r_{ij})T_{i}}.$$ (2)

**Fig 2:**
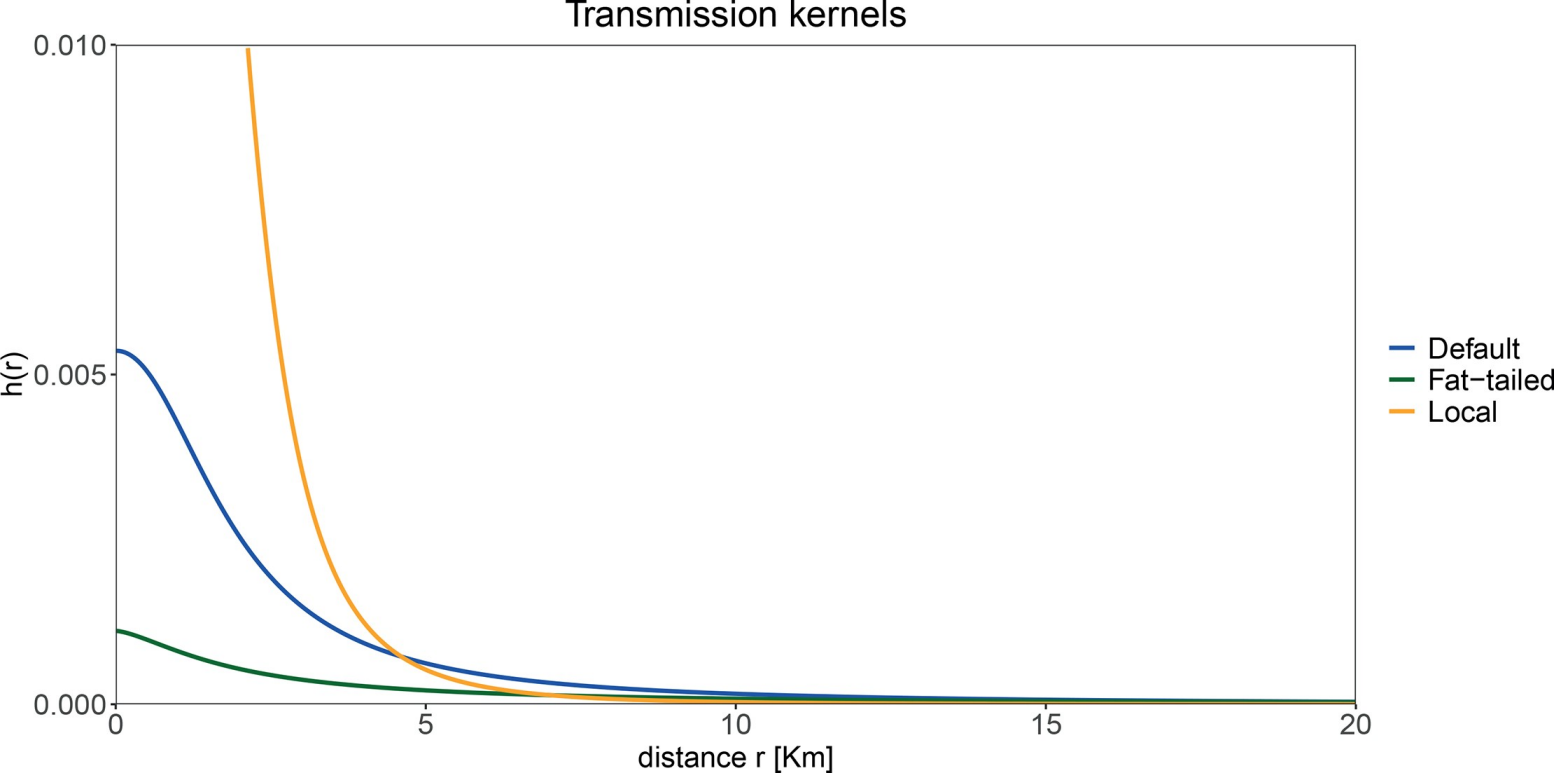
Transmission kernels. The parameter *h*_*0*_ has been rescaled such that *R*_*0*_, for an homogenous configuration, is the same for all the three kernels. Specifically, *R*_*0*_ is 1.9 for all three kernels; *α* = 1.5 and *h*_*0*_ = 0.001 for the fat-tailed kernel; *α* = 2,1 and *h*_*0*_ = 0.005 for the default kernel; *α* = 4 and *h*_*0*_ = 0.026 for the default kernel. Parameter values are based on estimates by Boender et al. [5] for the Dutch epidemic of avian influenza in 2003, but with rescaling to account for the decrease in number of farms (from 5,360 to 2,157) and concomitant increase in size of farms since 2003.

The function *h*(*r*_*ij*_) is called *transmission kernel* and it represents the hazard that infected host *i* exerts on susceptible host *j*. Throughout we use a transmission kernel of the shape:

$$h(r)=\frac{h_{0}}{1+{\left(\frac{r}{r_{0}}\right)}^{\alpha }},$$ (3)

where *h*_*0*_ is the hazard in the immediate vicinity of the infected host (*r* = 0), *r*_*0*_ is the distance at which the hazard is half of the maximal hazard, and *α* is the decay parameter which determines the shape of the kernel (Fig 2).

We simulate the model using a spatial Sellke construction [36–39]. Advantages of the Sellke construction over the better known Gillespie algorithm are that the method i) enables exact and efficient simulations of the epidemic [40], ii) is easily generalised, for instance by extension to non-exponentially distributed sojourn times, and iii) enables coupling of the epidemics on the same underlying probability space [41–43]. Such coupling of simulations facilitates the comparison of scenarios, as we will show below.

The Sellke construction keeps track of the cumulative infection pressure experienced by each susceptible host. Susceptible hosts are infected as soon as the cumulative infection pressure exceeds a stochastic threshold to infection *Q*_*j*_. In the case where *Q*_*j*_ *~ Exp*(1) the resulting epidemic corresponds to the SIR model, but the method can be generalized to other models and sojourn distributions [44]. The cumulative force of infection (*Λ*_*j*_(*t*)) exerted on an uninfected host *j* up to time *t* is given by

$$\Lambda_{j}(t)=\int_{0}^{t}\lambda_{j}(t\prime )dt\prime$$ (4)

where *λ*_*j*_(*t*) is the force of infection on susceptible host *j* at time *t* exerted by all the infected hosts at time t. Denoting the set of currently infected hosts by *I*, the force of infection on host *j* is given by:

$$\lambda_{j}(t)=\sum_{i∊I}h(r_{ij}),$$ (5)

with *h*(*r*) the transmission kernel defined in Eq (3). The Sellke construction has proved valuable not only for efficient simulation of epidemics, but also for theoretical advances in a variety of contexts [45–47].

Therefore, in the spatial transmission version of the Sellke construction, the probability of infection depends on the host’s individual threshold and on its proximity to infected host(s). Each infected host is characterized by an individual infectious period, which is drawn from a gamma distribution with shape and scale parameters *c* and *w* (mean = *cw;* variance *= cw*^*2*^). After the infectious period the host is not infectious anymore, it enters the removed state (*R*) where it does not contribute to the cumulative force of infection.

For each host *i* (*i = 1*,…,*N*), we can derive the individual reproduction number *R*_*i*_, which represent the expected numbers of secondary infections caused by an infected host at the start of the epidemic. When *R*_*i*_ >1, infection of the focal farm would cause on average more than one subsequent infection if all other farms in the vicinity were susceptible. When the infectious periods are drawn from a parametric distribution, *R*_*i*_ can be derived explicitly. In our case, the infectious periods *T* are drawn from a gamma distribution and *R*_*i*_ is given by [5]:

$$R_{i}=\sum_{j\neq i}\left\{1-{\left[\frac{1}{1+wh(r_{ij})}\right]}^{c}\right\}$$ (6)

### Risk mapping for epidemic transmission

We use the cumulative force of infection at the end of epidemics as a tool to compare epidemics. This is possible as the cumulative force of infection determines the probability that a host is infected given that the surrounding host(s) are infected. Specifically, the probability of infection is given by the cumulative distribution function of the cumulative force of infection, i.e. *p*(infection) = 1 − exp(-*Λ*).

**Fig 3:**
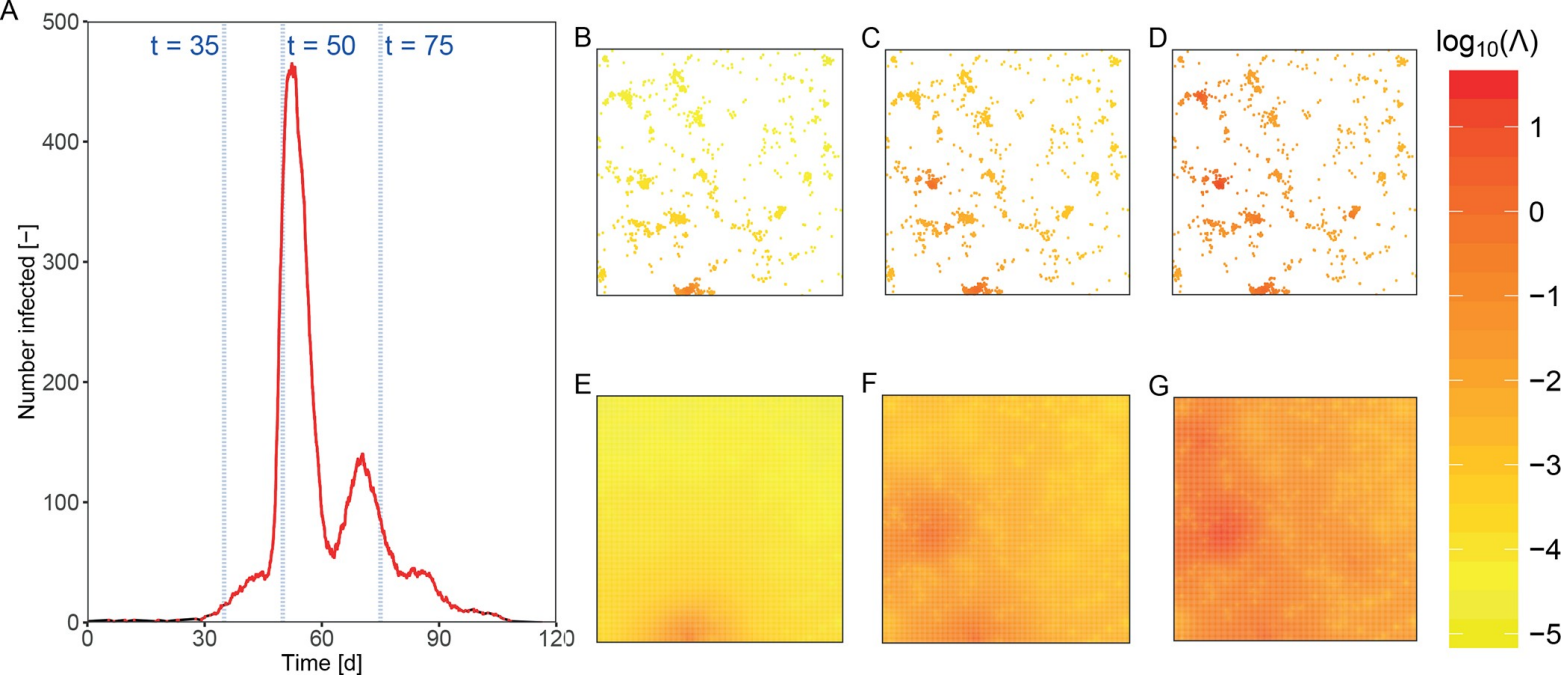
Temporal and spatial development of an epidemic in a clustered population and corresponding cumulative force of infection (*Λ*). A) Number of infected hosts over time. B-D) Map of the cumulative force of infection (in logarithmic scale) at three points in time, respectively *t* = 35 days, *t* = 50 days; *t* = 75 days. For each point host, the force of infection cumulated until that time point is plotted. E-G) Map of the interpolated cumulative force of infection (in logarithmic scale) at three time points. The pointwise cumulative force of infection (panels B-D) has been interpolated in a grid of 201 km by 201 km (grid resolution 1x1 km) by using inverse distance interpolation [48].

As an illustration, we simulate an epidemic in a clustered population, and plot the spatial and temporal unfolding of the cumulative force of infection (Fig 3). The number of infected hosts slowly increases at the beginning of the epidemic (Fig 3A) in the vicinity of an initially infected farm. As a consequence, the corresponding cumulative force of infection is high only in the areas surrounding the cluster of infected hosts (purple red spot at the bottom of Fig 3B and 3E). The sudden increases in the numbers of infected hosts at approximately *t = 45* and *t = 65* mark the time points when the infection hits new densely populated clusters of hosts. In this particular simulation, a significant fraction of hosts in dense clusters is ultimately infected, and the cumulative force of infection (and hence probability of infection) is non-negligible everywhere on the grid.

### Spatial clustering and transmission range determine epidemic size

We use the parameters estimated earlier for the epidemic of avian influenza in the Netherlands in 2003 to parameterize our default transmission kernel (blue line in Fig 2) [5]. We run this model 2,000 times for each of the 25 spatial patterns (Supporting Information for details). For each spatial pattern, every host is selected exactly once as the initial infective. To obtain a visualisation of the impact of spatial structure we average the cumulative force of infection at the end of epidemics over the 2,000 epidemics. From this we construct a risk map where the colours represent the expected probability of infection at a given location if the epidemic would start with a randomly selected host.

**Fig 4:**
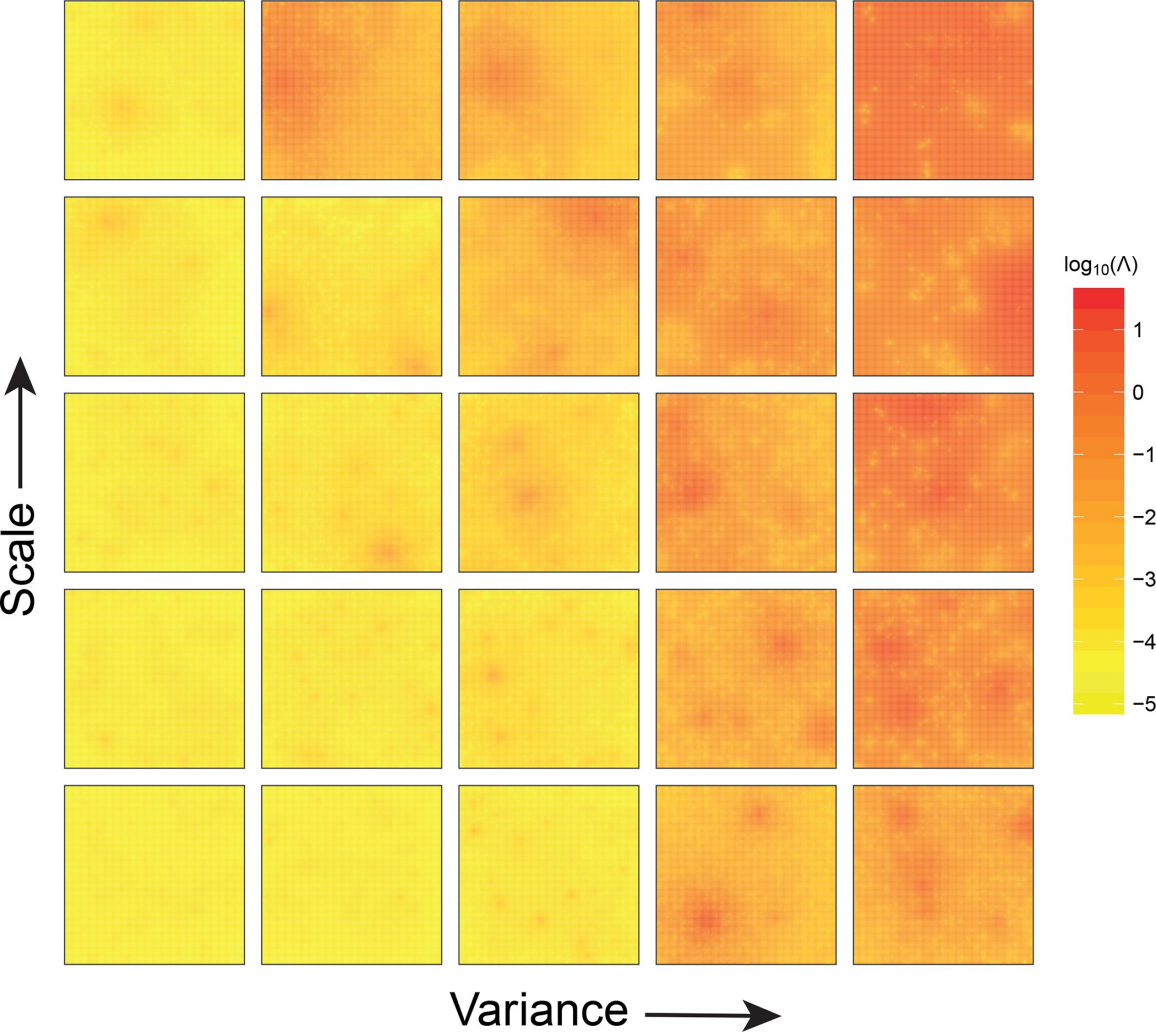
Risk maps for the 25 patterns of points characterized by a different level of clustering. Colour codes indicate the interpolated cumulative force of infection (*Λ*) at the end of the epidemic (logarithmic scale). The cumulative force of infection is averaged over 2,000 simulations.

In Fig 4, we show risk maps across the 25 different spatial point patterns of Fig 1. The cumulative force of infection shows little spatial variation in homogenous populations (Fig 4, bottom left) and in populations with high degree of clustering (Fig 4, top right). In the former, the probabilities of occurrence of a large epidemic are small (as indicated by low cumulative force of infection) and infections mostly occur to the vicinity of the site of introduction of the infection. In the latter, epidemics are large, and the probabilities of infection are high everywhere. Notice that this is true not only in the densely populated areas but also in areas with low density of hosts. This is the result of very high levels of transmission in the densely populated areas once they are hit.

In populations with intermediate levels of clustering the cumulative force of infection is variable, and is highest in larger areas with high density of hosts (Fig 4, middle panels). Overall, higher values of cumulative force of infection are observed with an increase in variance (moving from left to right in Fig 4). This is because an increase in variance yields more clusters with high density of hosts, facilitating epidemic transmission. For low values of the scale parameters, however, these clusters are like isolated hot spots with very high density concentrated in a very small area, thereby impeding cluster-to-cluster transmission (bottom right hand side of Fig 4).

**Fig 5:**
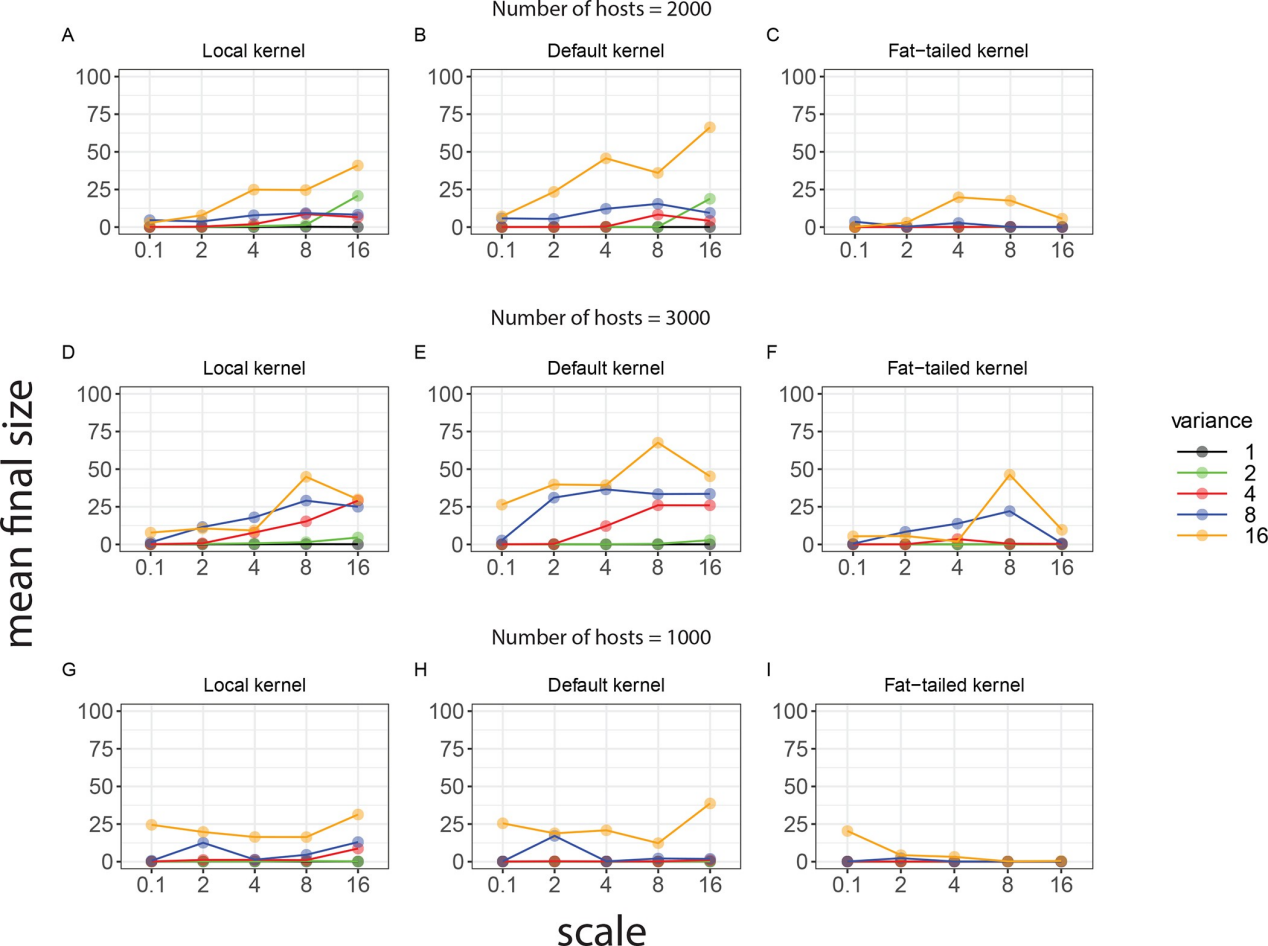
Means of the final size (expressed as percentage) plotted as a function of the scale (horizontal axis) and the variance of the random field (different curves) that generated the different patterns. The line plots are obtained by applying the model with: A) α = 4, *h_0_* = 0.026 (local kernel); B) α = 2.1, *h_0_* = 0.005 (default kernel); C) α = 1.5, *h_0_* = 0.001 (fat tailed kernel) on the point patterns with 2000 hosts shown in Fig 1. Panels D-F show results of simulations for the three different kernels described above, performed on 25 new point patterns with 3000 hosts (high density). Panels G-I show results of simulations for the three different kernels described above, performed on 25 new point patterns with 1000 hosts (low density).

Subsequently, we investigate how the sizes of epidemics depend on the dispersal characteristic of the infection. To this purpose, we use a “local” and a “fat-tailed” kernel while keeping overall transmissibility constant among scenarios (orange and green lines in Fig 2). For each scenario, we run the model 2,000 times for the 25 spatial point patterns by starting each time at a different location. The mean final size (i.e. the number of infected hosts) (Fig 5A–5C) is generally higher in clustered than in homogenously populations, independently of the characteristics of the transmission kernel.

Using fat-tailed kernels (Fig 5C) the probability of dispersion along long distances is relatively high and it would allow, in aggregated populations, for transmission among clusters (metapopulation dynamics). However, in such cases the individual reproduction numbers inside the cluster are often lower than 1 and the epidemic cannot take off within the cluster. In contrast, when dispersal is highly localized (Fig 5A), the final size is high inside the clusters but metapopulation dynamics seldom occur. This also explains why the mean final size of an epidemic is relatively low in case of a local kernel (Fig 5A). For most levels of clustering the default kernel (Fig 5B) appears to provide an optimal compromise between these opposing demands: on the one hand there is substantial within-cluster epidemic transmission, while on the other hand the probabilities of cluster-to-cluster transmission are still substantial, yielding higher mean final size than in the two extreme scenarios (Fig 5B versus Fig 5A and 5C). The exception to this rule is when the variance parameter is low and the scale parameter is high. In this case, the mean final size is highest for the local kernel.

We also investigate to what extent the aforementioned trade-off between localization and dispersion depends on the density of hosts. To this purpose, we generate 25 new point patterns with 3,000 hosts (high density) and 25 with 1,000 hosts (low density). For high density of hosts, the mean final size (Fig 5D–5F), is highest in the case of the default kernel especially for high values of the scale and variance. However, for low density of hosts (Fig 5G–5I) the mean final size of the epidemic is highest for the local kernel. This result shows that the trade-off between local transmission and long-range transmission also depends on the density of the hosts.

### The outbreak size in populations with strong clustering

**Fig 6:**
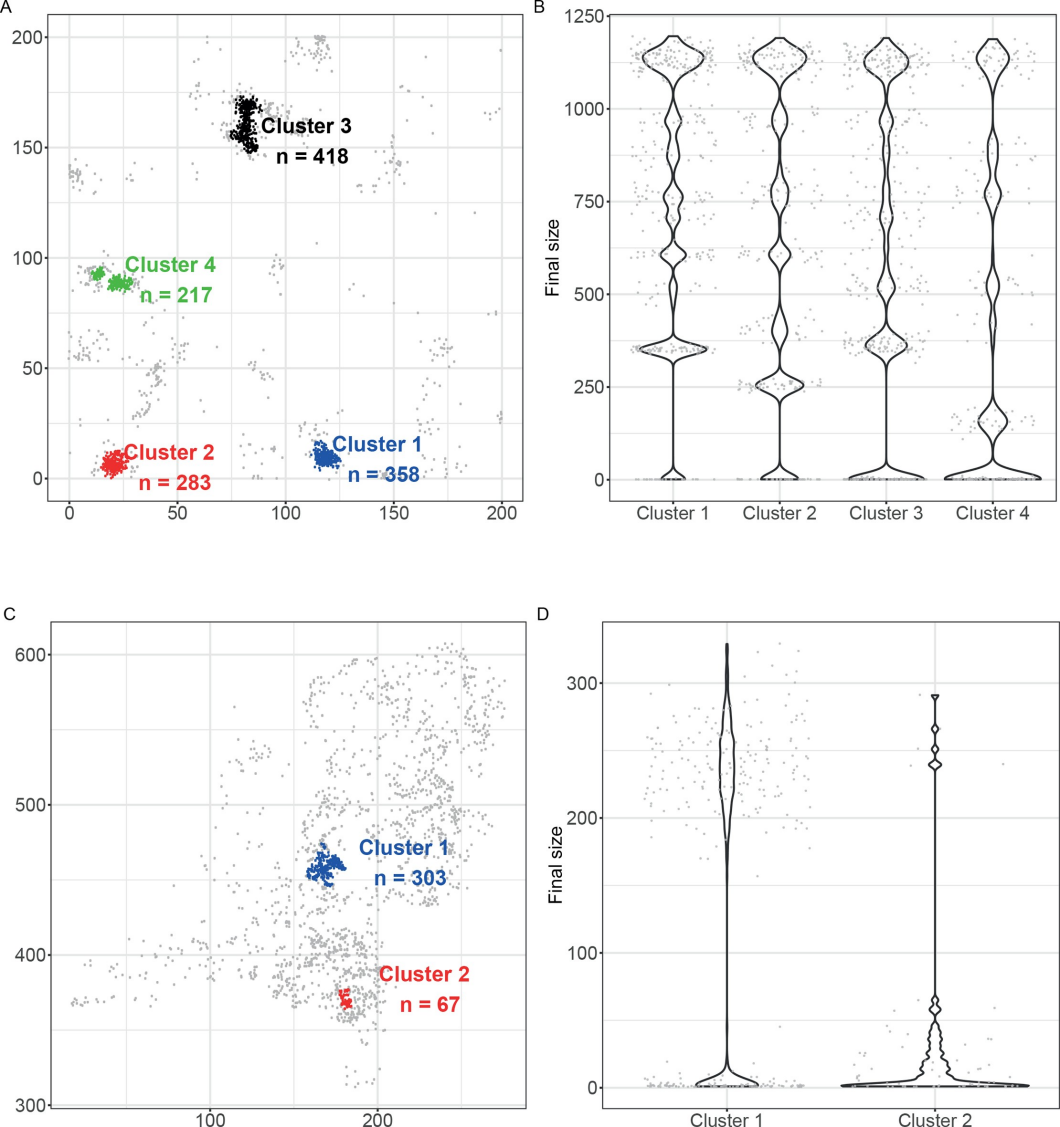
Distributions of outbreak sizes in clustered populations. A) Point pattern characterized by four high density clusters. The point pattern is generated by a random field with variance = 8 and scale = 8 (Supporting Information). B**)** Distributions of outbreaks sizes stratified by origin of the epidemic obtained by running the model in the point pattern shown in panel A. C) Map of Dutch poultry farms with more than 100 chickens. The point pattern is characterized by two clusters D) Distributions of outbreaks sizes stratified by origin of the epidemic obtained by running the model among the Dutch farms. Hosts in the clusters have individual reproduction number *R*_*i*_ >1, while hosts outside the clusters have individual reproduction number *R*_*i*_ ≥ 1 (Supporting Information). The spatial SIR model with default kernel has been run as many times as the number of hosts (2000 for panels A and B; 2175 for panels C and D, starting each time in a different host.

In populations with strong clustering the epidemic size is determined by the clusters of hosts where epidemic transmission is possible, and by the locations of these clusters. Fig 6A gives an example, using the default kernel. In the example there are four high-density clusters. In each of these, the individual reproduction numbers exceed the threshold value 1, while hosts in the surrounding area all have reproduction number below 1 (see Supporting Information for details). As in the previous examples, we run 2,000 simulations each time seeding the outbreak in a different host. We then computed the outbreak size of each simulation, stratified by cluster of origin (Fig 6).

For each of the high-density clusters the outbreak size is often much larger than the cluster size, while epidemics that are seeded in the low-density areas often remain small, indicating metapopulation-like dynamics where cluster-to-cluster transmission occurs frequently. Notice furthermore that the probability of a small outbreak in a cluster is inversely related to the cluster means of the individual reproduction numbers, and that even in major outbreaks that affect all clusters the outbreaks size is always smaller than the number of hosts in the cluster (range of outbreak sizes: 1,050–1,200; total number of hosts in clusters: 1,276) (Fig 6B; Table 1, Table A in S1 Supporting Information). Table 1 shows the total epidemic size for each cluster of origin, and Table A in S1 Supporting Information provides summary statistics of the high-density clusters, in particular the probabilities of a major outbreak, and the probabilities that an introduction in a given cluster results in a major outbreak in each of the other clusters. Together, Fig 6 and Table 1 and A in S1 Supporting Information illustrate that the transmission dynamics is characterised by epidemic transmission in areas with high density of hosts, hardly any onward transmission in areas with low density of hosts, and stochastic transmission from highly infected areas to densely populated areas that are as yet uninfected.

**Table 1:** Total epidemic size of the simulations and metapopulation approximation in a highly clustered population (Fig 6A). The total epidemic size for each cluster of origin is calculated by summing upon all clusters the product of the mean outbreak size and the probability of a major outbreak in each cluster (see values in Table A in S1 Supporting Information). Notice the fair correspondence between simulations and metapopulation approximation.

		*Simulation*	*Metapopulation approximation*
*Seeding cluster*	*n*	*Total epidemic size*	*Total epidemic size*
*1*	*358*	*614*	*689*
*2*	*283*	*533*	*608*
*3*	*418*	*457*	*558*
*4*	*217*	*344*	*450*

In the following we provide a numerical approximation to the final size in populations characterized by metapopulation-like dynamics, i.e. with high intensity of transmission within clusters and hardly any transmission to and from hosts in areas outside the main clusters. To do so we make a number of simplifying assumptions, mainly on independence between hosts, and use readily available theory on the probability of a major outbreak and size of a major outbreak. In the approximation, subpopulations are defined by local clusters of hosts with individual reproduction numbers exceeding the threshold value 1. The approximation contains the following steps:

#### 1) The probability of local extinction

Upon introduction of the infection in a cluster where epidemic transmission can occur, it is possible that the transmission chain gets stuck in the first few infection generations. The probability that this occurs can be calculated using branching process theory, and in our case is given by *P*(outbreak) = 1−*q*, where *q* is the solution in (0,1) of the equation *q* = *g*(*f*) and *g*(*f*) is the probability generating function (pgf) of the offspring distribution [38, 49]. In our case, no explicit formula exists for the pgf of the offspring distribution, but approximations are available. We use an approximation based on a gamma distributed individual reproduction numbers. In this case, the offspring distribution is negative binomial, and we have

$$g(f)={\left(1+\frac{R_{0}}{k}(1-f)\right)}^{-k},$$ (7)

where *R*_0_ = *E*(*R*_*i*_) is the mean of the individual reproduction numbers in clusters, and $k=\frac{R_{0}^{2}}{Var(R_{i})}$ is the corresponding dispersion parameter. Table A in S1 Supporting Information shows the results for the example of Fig 6A. We here used the methods of moments matching the means and variances of the gamma distributions with the means and variances of the individual reproduction numbers (see S1 Text and github repository available at https://github.com/elisabeninca/spatial_modelling)

#### 2) *The expected outbreak size in clusters*

With the individual reproduction numbers at hand, we obtain an approximate final size relation as follows. We assume that the probability *z*_*i*_ that host *i* is infected by transmission within the cluster can be approximated by $z_{i}=1-e^{-R_{i}\bar{z}}$ [50]. In essence this amounts to assuming that the hosts are independent (which they are not). Summing the final size equation over all hosts *i* in a cluster *I* (*i*∈*I*) and dividing by *n*_*I*_, the total number of hosts in cluster *I*, yields a final size equation for the expected fraction of hosts that is infected [50, 51]

$$\bar{z}=1-\frac{1}{n_{I}}\sum_{i=1}^{n_{I}}e^{-R_{i}\;\bar{z}}.$$ (8)

For given *R*_*i*_’s this equation is readily solved for the epidemic size $\bar{z}$, and subsequently by insertion also for the individual *z*_*i*_. These calculations form the basis of the results presented in Table 1, Tables A and B in S1 Supporting Information. An alternative approximation based on the assumption that individual reproduction numbers are independently gamma distributed yields quantitatively very similar results (S1 Text).

#### 3) *The probability of direct cluster-to-cluster transmission*

Assuming that areas between clusters are sparsely populated, we approximate the probability that an introduction in cluster *I* gives rise to a large outbreak in cluster *J* by direct transmission using the probabilities of local extinction (Eq (7)), the expected outbreak size in the cluster of origin ($n_{J}\;{\bar{z}}_{J}$) (Eq (8)), the distance between the centres of gravity of the clusters (*r*_*IJ*_), and the expected number of infections in cluster *J* caused by an infection in cluster *I* (*R*_*IJ*_(*r*_*IJ*_); Eq (6)). The total hazard presented by an introduction in cluster *I* to cluster *J* is then given by

$$H_{IJ}=(1-q_{J})\;R_{IJ}(r_{IJ})\;n_{I}\;{\bar{z}}_{I}\;(1-q_{I}),$$ (9)

and the probability that an introduction in cluster *I* gives rise to a large outbreak in cluster *J* is then given by:

$$p_{IJ}=1-e^{-H_{IJ}}.$$ (10)

Hence, the probability of direct cluster to cluster transmission can, under certain assumptions, be approximated using standard epidemiological theory.

#### 4) The overall probability of cluster to cluster transmission

Ultimately, what matters is the probability that a cluster *J* is infected either directly or indirectly and that a major outbreak ensues. In case of the four clusters in the example, the overall probabilities of transmission are readily calculated using all direct, one-step, and two-step cluster-to-cluster transmission routes. By comparing the observed simulated probabilities that an introduction in a cluster *I* yields a major outbreak in cluster *J* with the corresponding calculated probabilities we find that in the example of Fig 6A and 6B, the above calculations usually give a reasonable approximation. In fact, the mean absolute and relative errors are 0.035 and 0.073, respectively (Supporting Information).

### Metapopulation dynamics among poultry farms in The Netherlands

We apply the approach in the above example (Fig 6A and 6B) to the real-world example of avian influenza transmission between poultry farms in The Netherlands (Supporting Information for details). We define the clusters by applying the same criterion as before (Fig 6C) to define the clusters where epidemic transmission is possible. Two main clusters are identified: one in the centre of the country, the intensively farmed area called Gelderse Vallei and one in the south-east of the country (Fig 6C). The final size of epidemics is for both clusters almost always smaller than the cluster size (Fig 6D), indicating that cluster-to-cluster transmission is rare (Table B in S1 Supporting Information). However, in the rare cases that cluster-to-cluster transmission does occur, it can have major impact, as shown by the (infrequent) transmission events from the smaller cluster 2 to the larger cluster 1 (Fig 6D). In contrast with the example of Fig 6A (Table 1, Table A in S1 Supporting Information), the metapopulation model does not provide a good approximation of the total epidemic size in case of avian influenza transmitted between poultry farms in the Netherlands, indicating that non-negligible transmission occurs outside the two clusters.

## Discussion

In this study, we systematically analysed how the transmission of infectious diseases between immobile hosts is determined by the interplay between host clustering and the spatial range of the transmission kernel. Our analysis combined spatial process and transmission modelling, using epidemic size as overall measure of transmission and using the local reproduction number and probability of infection as measures to characterize the spatial variation in transmission. We find that for a given degree of clustering and individual-level infectivity, the total number of infections is maximal if the range of transmission is intermediate. Our results thus complement and extend the findings of Brown and Bolker (19) that the epidemic threshold can be maximal at intermediate transmission range and which is due to a trade-off between local and distant transmission. In addition, we show that this trade-off also depends on the density of hosts, being absent at low densities and being strong at high densities. This has practical implications, for instance for epidemics in crops or between farms in densely populated areas. Furthermore we find that in highly clustered populations, the infection dynamics is strongly determined by the probability of transmission between clusters of hosts, whereby local clusters act as multiplier of infection. We show that in this regime, a metapopulation model of the clusters can sometimes provide a good approximation of the total epidemic size. This metapopulation approximation is reminiscent of an earlier approach [52] and uses as building blocks the probabilities of local extinction, the final size in clusters, and probabilities of cluster-to-cluster transmission. The implication is that in the highly clustered regime, the transmission dynamics between hosts can be viewed as in essence being determined by transmissions between clusters. The real-world example of avian influenza transmission between poultry farms in the Netherlands shows that the highly clustered regime is relevant in practice and that the metapopulation approximation provides additional insight into how the within-cluster and between-cluster transmission risks together shape the final size distribution.

Regarding the overall probability of sustained transmission upon an introduction, we find that it is low in homogenous populations and high in clustered populations. This is in agreement with earlier results [19], in which the effect of spatial clustering on the epidemic threshold was analysed. In addition, we observe that the probability of continued transmission is generally higher for higher values of the variance parameter generating the distribution of hosts. Our interpretation is that this is because in fields with high variance the probability of early extinction is low, as most introductions are in dense (albeit possibly small) clusters.

The analysis presented here focuses mainly on one aspect of the transmission dynamics, namely the final size of the epidemics. However, previous studies have shown that the rate of dispersal of the infection might also affect the duration of the epidemics, i.e. high dispersal can lead to shorter epidemics in the context of non-sessile hosts [53]. Other studies have shown that the duration of epidemics might be influenced by the spatial distribution of hosts [54], although the relationship is still poorly understood. Studies with experimental plant population of hosts [55] showed that in clustered host populations, epidemics unfolded more quickly at first, then later more slowly, than in hosts with uniform host distributions. It is therefore plausible that the interplay between the rate of dispersal (e.g. the shape of the dispersal kernel) and the spatial distribution of hosts also affects the duration of epidemics. This is at the present still an open question.

In the analysis, we assumed for simplicity that the transmission kernel depends only on the Euclidean distance between hosts. In fact, the development of models with realistic non-isotropic distance-based kernels remains a big challenge [56]. This is perhaps even truer in cases where transmission cannot be described with a distance-based function. For instance, our results do not provide insight when transmission is determined by a trade network, and instead trade network models would need to be employed [57–59].

Our initial aim was to develop a method that could a priori identify patterns of hosts that would present a risk for sustained transmission after an introduction. This would be of great practical relevance, for instance, in cases where an estimate (or guess) of the spatial range and intensity of transmission would be available, but outbreaks would not (yet) have been observed. A prominent example is the evaluation of the risk posed by introductions of avian influenza in poultry, which in practice is based on just a handful of actual outbreaks [5, 60]. Fortunately, methods are available to estimate characteristics of spatial point patterns [31], which could enable an a priori assessment of risk of epidemic transmission if estimates of the scale and variance parameters would be available.

In addition, we believe that such approaches could aid determining the critical vaccination coverage in populations with strong clustering of susceptible hosts [61].

## Supporting information

S1 Supporting Information Supplementary methods, tables and figures. (PDF)

S1 Text R code and Mathematica code. (PDF)
