# Supplementary material for: Trade-off between local transmission and long-range dispersal drives infectious disease outbreak size in spatially structured populations
Source: PLoS Comput Biol. 2020 Jul 6;16(7):e1008009. doi: 10.1371/journal.pcbi.1008009 (PMC7365471; doi:10.1371/journal.pcbi.1008009)
Supplement: S1 Supporting Information — (PDF) [file pcbi.1008009.s001.pdf]

Supporting Information: Trade-off between local transmission and long-range dispersal drives infectious disease outbreak size in spatially structured populations

Elisa Benincà[1*], Thomas Hagenaars[2], Gert Jan Boender[2], Jan van de Kassteele[1], Michiel van Boven[1]

[1]Centre for Infectious Disease Control, National Institute for Public Health and the Environment, 3720 BA Bilthoven, The Netherlands

[2]Department of Bacteriology and Epidemiology, Wageningen Bioveterinary Research, 8200 AB Lelystad, The Netherlands

*elisa.beninca@rivm.nl

Supplementary methods

Spatial Transmission Model

We run the model 2,000 times for each of the 25 patterns of hosts, starting each time the epidemic in a different location such that at the end of the 2,000 runs all 2,000 hosts have been used as starting point of the epidemic. For each of the 2,000 hosts, we draw in advance from a gamma distribution (c=10; w=7/10) its characteristic infectious period. We repeated this procedure 2,000 times, as the number of simulations, and we store the data in a matrix of 2,000 x 2,000. We repeat the same procedure for the individual thresholds Q_i. For each of the 2,000 hosts, we draw the individual threshold from an exponential distribution of mean 1, we repeated the procedure 2,000 times and we store them in another matrix of 2,000 x 2,000. We used these matrices for all patterns. This approach is particularly useful because it allows easy comparison of results among point patterns and therefore it makes easy to assess how the cluster structure affects the dynamics. Moreover, it also allows an easy comparison of the effects of different kernels on the same pattern of hosts. The interested reader is referred to the literature for excellent examples of the application of the Sellke construction [1-3].

As a starting point we used the parameters estimated by Boender et al. [4] for the Dutch epidemic of avian influenza in 2003. To evaluate the effect of dispersal, we changed the shape of the kernel by varying the parameter α. In order to fairly compare the effect of the three kernels, the R_0 for a homogeneous pattern should be the same for the three different kernels. In a homogenous pattern with constant density of points ρ, R_0 is the same everywhere and can be approximated to:

$$R_0 = 2\pi\rho\int_0^{\infty}\left(1-\exp\left(\frac{h_0}{1+\left(\frac{r}{r_0}\right)^{\propto}}\right)\right)rdr \qquad \text{(S1)}$$

For small values of h_0, as the ones used in this paper, this can approximated by:

$$R_0 = 2\pi\rho h_0 \int_0^{\infty} \left(1 - \exp(\frac{1}{1+\left(\frac{r}{r_0}\right)^{\propto}})\right) rdr \qquad \text{(S2)}$$

We now consider two kernels $h_1(r)$ and $h_2(r)$ with parameters respectively α_1, h_{01} and α_2, h_{02}.

R_0 is the same for the two kernels when:

$$h_{02} = \frac{h_{01} \int_0^{\infty} \left(1 - \exp(\frac{1}{1+\left(\frac{r}{r_0}\right)^{\propto 1}})\right) rdr}{\int_0^{\infty} \left(1 - \exp(\frac{1}{1+\left(\frac{r}{r_0}\right)^{\propto 2}})\right) rdr} \qquad \text{(S3)}$$

We rescaled the local and the fat-tailed kernels as a function of the default kernel by applying eq. *S3*. Because the integral for alpha < 2 is infinite, we approximated the integral in a radius of 200 km for the rescaling of both kernels. The rescaled local and fat-tailed kernels are shown in Fig 1 of the main text.

Definition of clusters

In the metapopulation model we defined a cluster as the pattern of points meeting the following two criteria:

1) For practical considerations, points are included in an area with a radius of 25 km around the point with the maximum local density. This procedure yields high density clusters that are clearly separated.
2) The points are characterized by an individual reproduction number R_i >1.

The local density of each point farm was estimated for the pattern of points (Fig 6A) and for the poultry farms in the Netherlands (Fig 6C) by fitting an isotropic Gaussian kernel. The variance of the kernel is calculated by using the function density.ppp provided in the Rpackage spatstat. The function computes a kernel smoothed intensity from a point patterns and it assigns a (local) density at each point of the pattern (see example in Fig C in S1 Supporting Information). The variance of the smoothed kernel is evaluated according to the Diggle algorithm [5, 6] by using the option bw.diggle. For the analysis of the metapopulation dynamics among the poultry farms in the Netherlands, we used the transmission model with the default kernel (α = 2.1) estimated by Boender et al. [4]. Since 2003 the numbers of farms has significantly decreased but the number of poultry per farm has significantly increased. To reproduce a situation comparable to the epidemic of 2003, we then set

the value of h_0 to 0.008 to obtain approximately the same percentage of farms with $R_0>1$ of the epidemic of 2003 (17%).

References

1. House T, Ross JV, Sirl D. How big is an outbreak likely to be? Methods for epidemic final-size calculation. Proc R Soc A. 2013;469(2150):20120436.
2. Cook A, Gibson G, Gottwald T, Gilligan C. Constructing the effect of alternative intervention strategies on historic epidemics. Journal of the Royal Society Interface. 2008;5(27):1203-13.
3. Brand SP, Tildesley MJ, Keeling MJ. Rapid simulation of spatial epidemics: A spectral method. Journal of theoretical biology. 2015;370:121-34.
4. Boender GJ, Hagenaars TJ, Bouma A, Nodelijk G, Elbers AR, de Jong MC, et al. Risk maps for the spread of highly pathogenic avian influenza in poultry. PLoS Comput Biol. 2007;3(4):e71. Epub 2007/04/24. doi: 10.1371/journal.pcbi.0030071. PubMed PMID: 17447838; PubMed Central PMCID: PMCPMC1853123.
5. Diggle P. Statistical Analysis of Spatial Point Patterns. 2nd Edition ed: Arnold; 2003.
6. Berman M, Diggle P. Estimating weighted integrals of the second-order intensity of a spatial point process. Journal of the Royal Statistical Society Series B (Methodological). 1989:81-92.

Supplementary tables

				Simulation						*Metapopulation approximation*					
Seeding cluster	*n*	$R_0 = E(R_i)$	*Var*(R_i)	*Probability of a major outbreak in the seeding cluster*	*Mean outbreak size in the seeding cluster*	*Probability of a major outbreak in cluster*				*Probability of a major outbreak in the seeding cluster*	*Mean outbreak size in the seeding cluster*	*Probability of a major outbreak in cluster*			
						1	*2*	*3*	*4*			*1*	*2*	*3*	*4*
1	*358*	*3.82*	*1.66*	*0.94*	*316*	*-*	*0.60*	*0.56*	*0.54*	*0.95*	*336*	*-*	*0.60*	*0.47*	*0.47*
2	*283*	*2.58*	*0.70*	*0.88*	*213*	*0.55*	*-*	*0.49*	*0.54*	*0.87*	*243*	*0.56*	*-*	*0.42*	*0.48*
3	*418*	*2.08*	*0.32*	*0.77*	*249*	*0.44*	*0.40*	*-*	*0.45*	*0.79*	*328*	*0.41*	*0.40*	*-*	*0.43*
4	*217*	*1.75*	*0.22*	*0.64*	*92*	*0.36*	*0.35*	*0.39*	*-*	*0.68*	*148*	*0.37*	*0.40*	*0.39*	*-*

Table A. Summary statistics of the simulation and metapopulation approximation in a highly clustered population (Fig 6A). Shown are the number of hosts in the cluster (n), the means and the variances of the individual reproduction numbers. Also shown are the probability of a large outbreak in the seeding cluster (for simulations operationally defined as >10 infections), the mean outbreak size in the seeding clusters, and the probability that the seeding results in a large outbreak in one of the other high density clusters, both for the simulations and the metapopulation approximation. Notice the fair correspondence between simulations and metapopulation approximation.

				Simulation				*Metapopulation approximation*			
Seeding Cluster	*n*	$R_0 = E(R_i)$	$Var(R_i)$	*Probability of a major outbreak in the seeding cluster*	*Mean outbreak size in the seeding cluster*	*Probability of a major outbreak in cluster*		*Probability of a major outbreak in the seeding cluster*	*Mean outbreak size in the seeding cluster*	*Probability of a major outbreak in cluster*	
						1	*2*			*1*	*2*
1	*302*	*1.71*	*0.17*	*0.61*	*116*	*-*	*0.075*	*0.67*	*202*	*-*	*0.056*
2	*63*	*1.24*	*0.03*	*0.30*	*7*	*0.076*	*-*	*0.36*	*23*	*0.031*	*-*

Table B. Summary statistics of the simulation and metapopulation approximation for the transmission dynamics among poultry farms in The Netherlands (Fig 6C). Shown are the number of hosts in the cluster (n), the means and the variances of the individual reproduction numbers. Also shown are the probability of a large outbreak in the seeding cluster (for simulations operationally defined as >10 infections), the mean outbreak size in the seeding clusters, and the probability that the seeding results in a large outbreak in one of the other high density clusters, both for the simulations and the metapopulation approximation. Notice the discrepancy in outbreak size with the simulations, especially with regard to the outbreak in the smaller cluster 2.

Supplementary figures

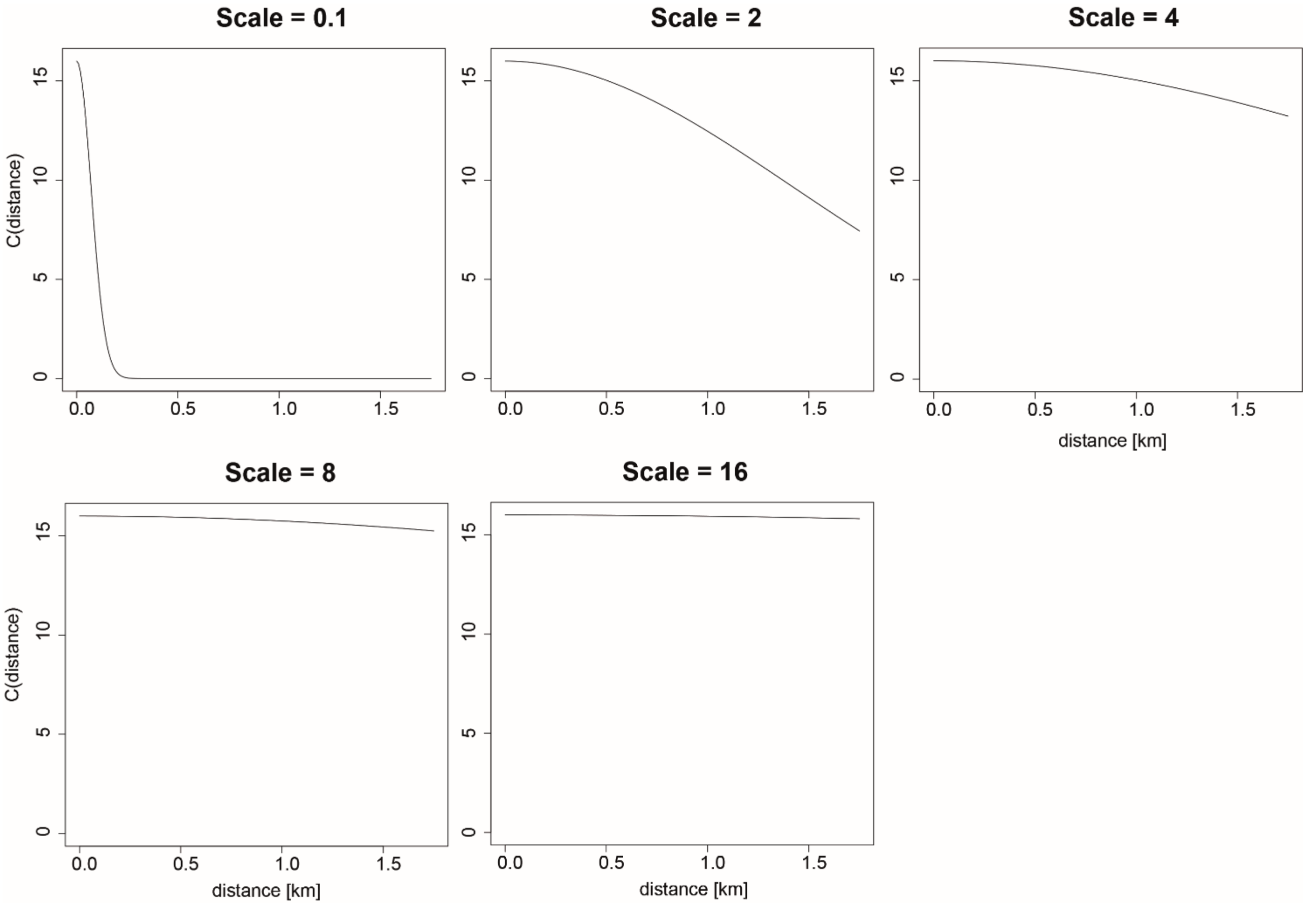

Figure A. Covariance as a function of the distance *r* between two points. The covariance function changes by varying the scale parameter *s* in the covariance model.

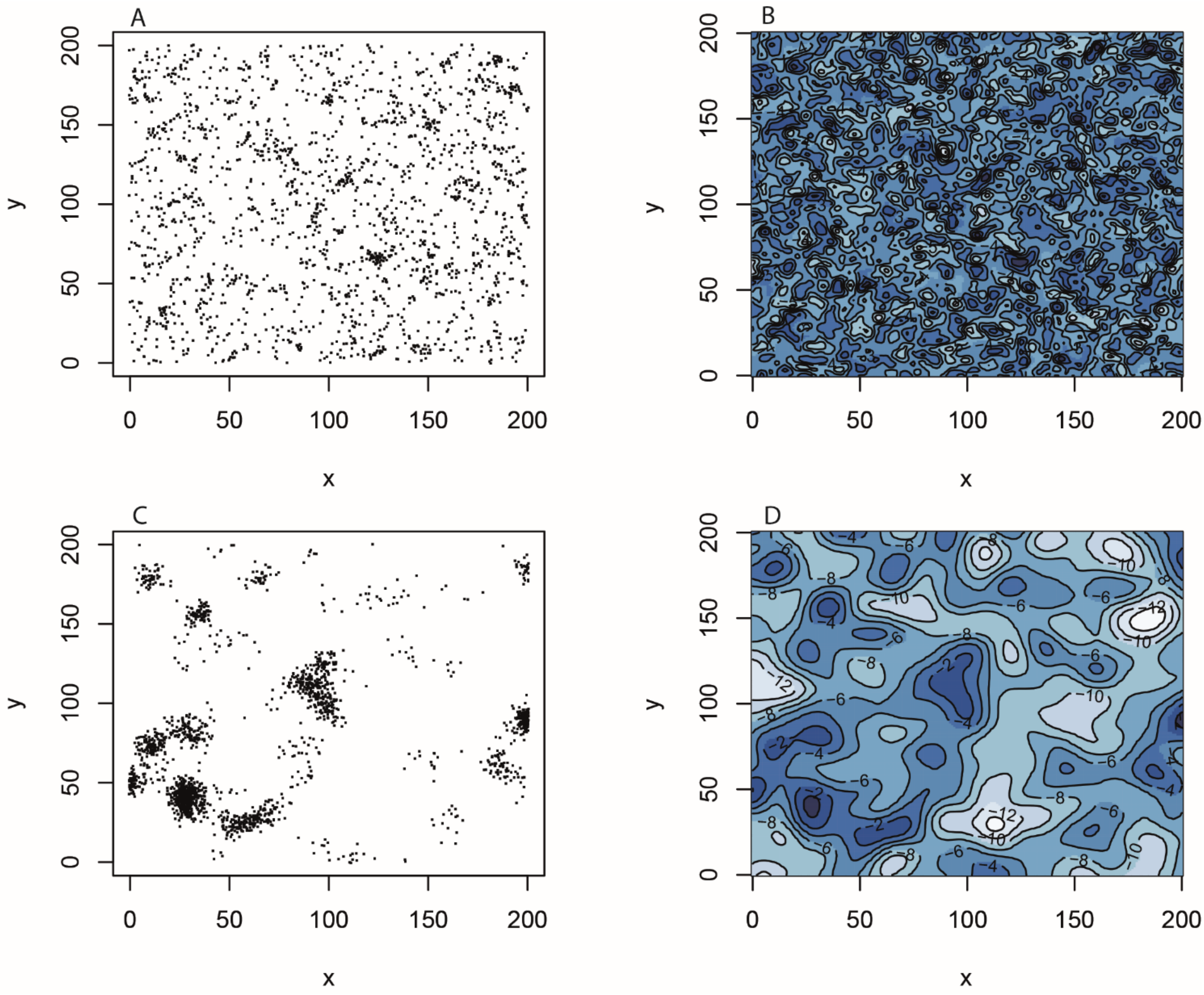

Figure B. Two examples of spatial point patterns of 2,000 farms and their corresponding random field. The spatial point patterns are obtained by sampling from a multinomial process with a probability equal to the intensity per grid unit of the realization of the corresponding random field divided by the sum of intensities of the entire grid. A) Spatial point pattern and B) the ln(intensity) of its corresponding realization of the random field obtained whilst imposing in the covariance model the scale parameter equal to 4 and the variance equal to 1.C) Spatial point pattern and D) the ln(intensity) of its corresponding realization of the random field obtained whilst imposing in the covariance model the scale parameter equal to 16 and the variance equal to 8.

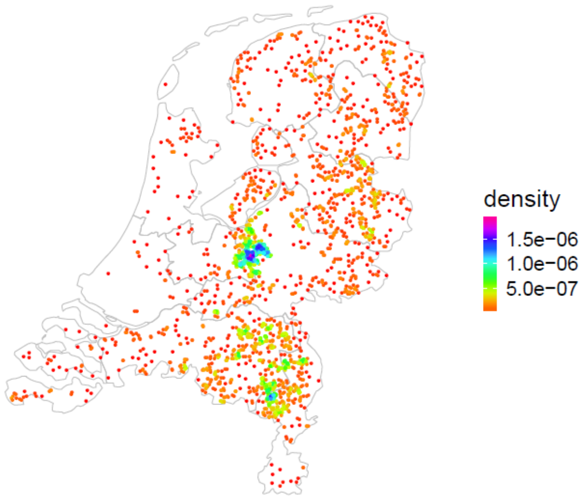

Figure C. Map of the local density of chickens farms in the Netherlands. Colour codes represent the local density of each farm. The local density for each farm is obtained by fitting an isotropic Gaussian kernel.

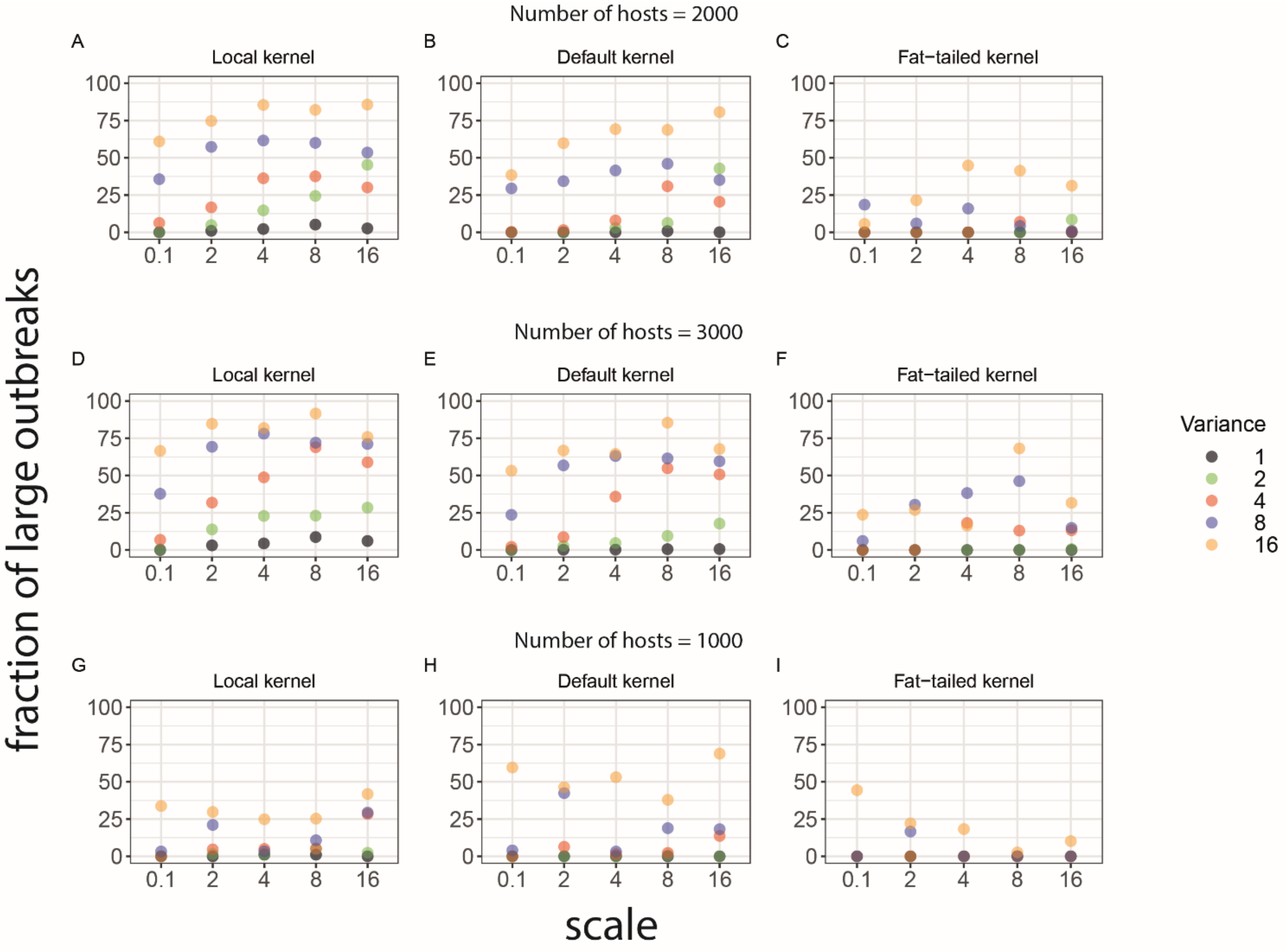

Figure D. Fraction (expressed as percentage) of simulations that yielded a large outbreak (operationally defined as outbreaks with at least 10 infections) plotted as function of the scale (horizontal axis) and the variance of the random field (different curves) that generated the different patterns. The plots are obtained by applying the model with: A) $\alpha=4$, $h_0=0.026$ (local kernel); B) $\alpha=2.1$, $h_0=0.005$ (default kernel); C) $\alpha=1.5$, $h_0=0.001$ (fat tailed kernel). Panels D-F show results of simulations for the three different kernels described above, performed on 25 new point patterns with 3000 hosts (high density). Panels G-I show results of simulations for the three different kernels described above, performed on 25 new point patterns with 1000 hosts (low density).

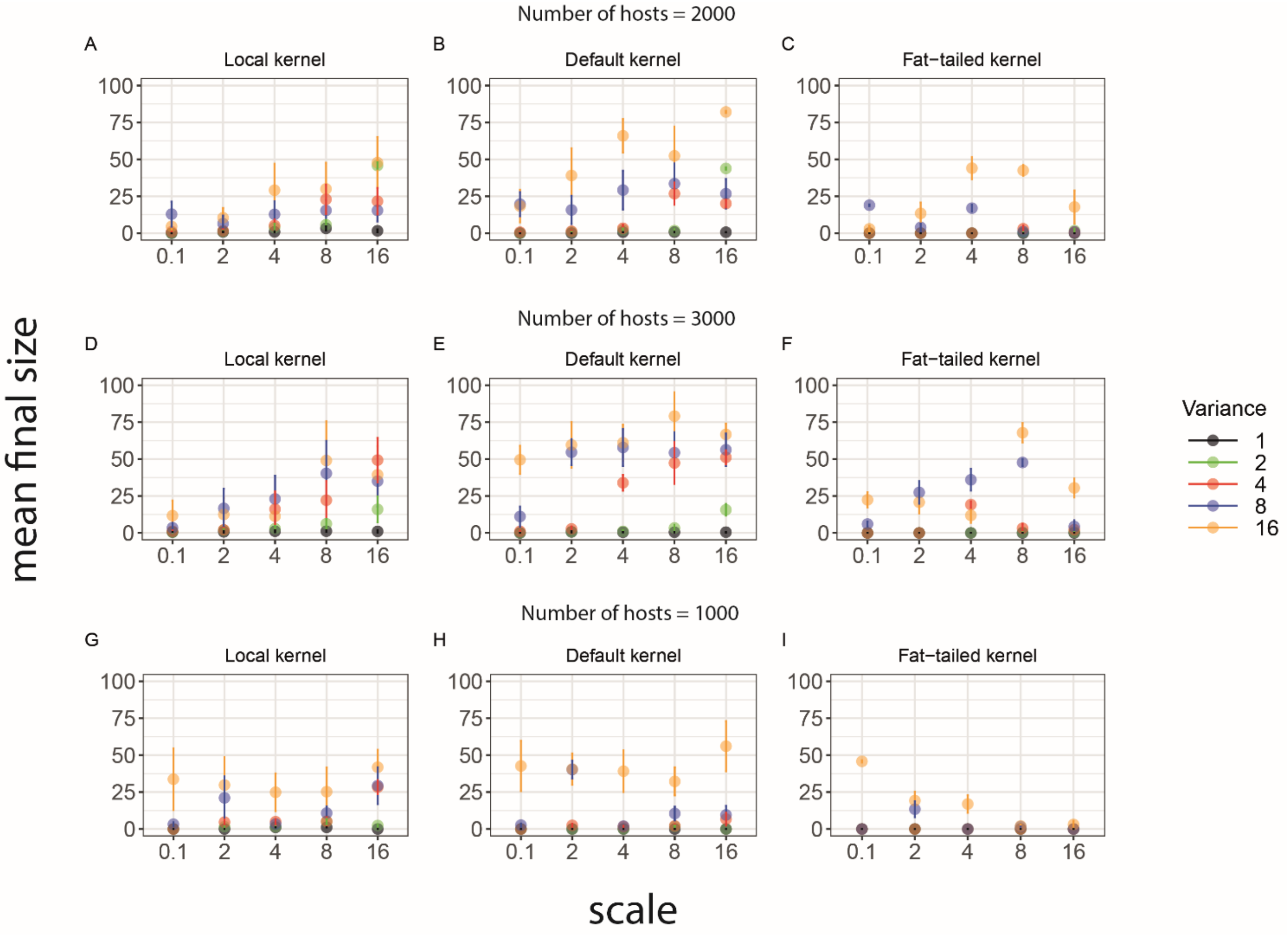

Figure E. Means and standard deviations of the final size (expressed as percentage) for major epidemics (i.e. final size exceeding 10 infections) plotted as function of the scale (horizontal axis) and the variance of the random field (different curves) that generated the different patterns. The plots are obtained by applying the model with: A) $\alpha=4$, $h_0=0.026$ (local kernel); B) $\alpha=2.1$, $h_0=0.005$ (default kernel); C) $\alpha=1.5$, $h_0=0.001$ (fat tailed kernel). Panels D-F show results of simulations for the three different kernels described above, performed on 25 new point patterns with 3000 hosts (high density). Panels G-I show results of simulations for the three different kernels described above, performed on 25 new point patterns with 1000 hosts (low density).

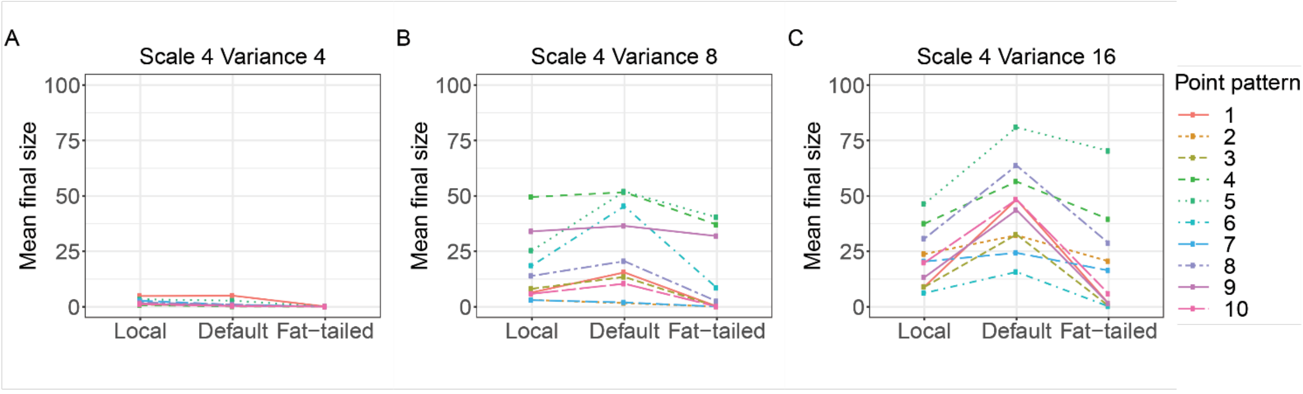

Figure F. Means of the final size (expressed as percentage) for different realizations (point patterns) of the random field. We chose three different pairs of parameters values of the random field: A) scale 4 and variance 4; B) scale 4 and variance 8; C) scale 4 and variance 16 and for each pair we generate 10 different points patterns of 2000 points. For each point pattern and for each of the three transmission kernels we run the model 2000 times and we calculated the mean final size. The mean value of the final size varies among point patterns. However, the general trend remains the same: the final size is higher for the default kernel compared to the other two kernels and this is particularly true for higher values of the variance parameter. Hence, the trade-off between local and long range transmission shown in the results of Fig 5 is independent on the point pattern chosen.
