# Supplementary material for: Trade-off between local transmission and long-range dispersal drives infectious disease outbreak size in spatially structured populations
Source: PLoS Comput Biol. 2020 Jul 6;16(7):e1008009. doi: 10.1371/journal.pcbi.1008009 (PMC7365471; doi:10.1371/journal.pcbi.1008009)
Supplement: S1 Text — (PDF) [file pcbi.1008009.s002.pdf]

Code

Here we provide the R code of the SIR spatial transmission model with the Sellke construction and the mathematica notebook of the metapopulation approximation.

These and other scripts used in the paper can also be found at

https://github.com/elisabeninca/spatial_modelling

R code of the SIR spatial transmission model with the Sellke construction.

```
# Spatial SIR transmission model with the Sellke construction
# The model simulates infections spread between immobile hosts (e.g. poultry
farms, plants) using the Sellke construction.
# The model assumes only removal of the detected hosts (i.e. no preventive
removal)

# Load the configuration of points and the matrices of Qinit and Tinf
load("configuration_points_200X200_whittle.Rdata")
# the matrices of Tinf and Qinit have been generated by the following code. To
use exactly the same matrices used in the paper load the matrices here
# numsim <-2000
# totpoints <- nrow(points_conf)
# Q_init_matrix <- matrix(0,nrow=numsim,ncol=totpoints)
# T_inf_matrix <- matrix(0,nrow=numsim,ncol=totpoints)
# for (ii in 1:numsim){
#  Q_init_matrix[ii,] <- rexp(totpoints, rate = 1)
#  T_inf_matrix[ii,] <- rgamma(totpoints,10, scale=7/10)
# }
load("TinfQinit.Rdata")
# Choose one of the point patterns
index<-27
# Select one point pattern (x and y coordinates) out of the 25. The matrix has 50
columns, because there are x and y coordinates for each point
matrix_points <- points_conf[,c(index,index+1)]  # I select one point pattern as
example
totpoints <- nrow(matrix_points) # total number of points
colnames(matrix_points) <- c("xcoord","ycoord")
# Add a column for the index
Index_points <- c(1:totpoints)
# Calculate the matrix of distances between points
# Express the coordinates as complex numbers for fast calculation of the
euclidean distance
Coord <-
(complex(length.out=2,real=matrix_points$xcoord,imaginary=matrix_points$ycoord));
distancematrix <- as.matrix(abs(outer(Coord,Coord,"-")))

# Define the transmission kernel and calculate the hazard matrix
# Rescale the parameters h0 used in Boender et al. to account for the change in
size and number of farms (see main text)
h0 <- 0.002*5360/totpoints;
alpha  <- 2.1;
r0  <- 1.9;
h_kernel <- function(r){h0/(1 + (r/r0)^alpha)} ; # transmission kernel as a
function of r
beta<-1;
# Create an hazard matrix evaluating for each host j the chance to be infected by
host i as a function of distance
```

```
hazardmatrix  <- as.matrix(apply(distancematrix,MARGIN=c(1,2),FUN=h_kernel));
diag(hazardmatrix) <- matrix(0,nrow=totpoints); # because the chance of infecting
itself is 0

# Model the transmission event between hosts
# Define the function handling the events in the spatial transmission model
# Event function has four entries: event_time= time at which an event occurs,
eventtype= type of event, statustype=status of host,id_= ID of host
# eventtype:  2=infection; 3=removal;
# statustype: 1= susceptible; 2= infectious; 3= culled;
event <- function(time_event,eventype,statustype,id_){
  if (eventype==2 & statustype==1){
    # calculate the CFI up to that moment
    # I let the CFI grow also for the infected hosts, because they are infected.
Of course, the already infected hosts will not be considered in the calculation
of the next infection time, because they are already infected
    CFI <<- CFI+
beta*apply(matrix(hazardmatrix[which(indexI==1),],nrow=length(which(indexI==1)),t
otpoints),   MARGIN=2,FUN=sum)*(time_event-tt)     # In the rows (i) the
infected, in the columns (j) the susceptibles
    # save the CFI in the matrix of CFI
    index_new_event <<- index_new_event + 1
    CFI_matrix[index_new_event,] <<- CFI
    timevector <<- rbind(timevector,time_event)
    # update the status vector and the indices vectors for S and for I
    Status[id_] <<- 2 # now Status= 2 (infected)
    indexI[id_] <<- 1 # infected
    indexS[id_] <<- 1 # not susceptible anymore
    # save the number of infected over time in a list
    infected_over_time <<-
rbind(infected_over_time,c(time_event,length(indexI[indexI==1])))
    # calculate the slope of the force of infection from this moment onwards
    bb[which(indexS==0)] <<-
beta*apply(matrix(hazardmatrix[which(indexI==1),which(indexS==0)],nrow=length(whi
ch(indexI==1)),ncol=length(which(indexS==0))), MARGIN=2,FUN=sum)
    # in the rows (i) the infected, in thecolumns (j) the susceptibles
    # calculate the next infection events
    t_infection[which(indexS==0)] <<- (Q_init[which(indexS==0)]-
CFI[which(indexS==0)])/bb[which(indexS==0)]
    t_infection[which(indexS==1|indexS==3)] <<- 10000000 # I set a very high
number which is not going to happen
    # update the list of points to infect
    next_infection_host <<- which.min(t_infection)
    next_infection_time <<- t_infection[which.min(t_infection)]
    #update the time
    tt <<- time_event
    #update the list of hosts to infect
    List_to_infect <<- rbind(List_to_infect,data.frame(Event_time =
tt+next_infection_time,   Type_event = rep(2,length(next_infection_host)),
id_host = next_infection_host))
    List_to_infect <<- List_to_infect[order(List_to_infect[,1]),]
    #update the list of hosts to remove
    List_to_remove <<-
rbind(List_to_remove,data.frame(Event_time=tt+T_inf[id_],Type_event=3,id_host=id_
))
    List_to_remove <<- List_to_remove[order(List_to_remove[,1]),]
    return(1)} else if (eventype==2 & statustype==2) {# already infected
      return(0)} else if (eventype==2 & statustype==3) {# if it has been culled
it cannot be infected
        return(0)} else if (eventype==3 & statustype==1) {# it does not occur
          return(0)}else if (eventype==3 & statustype==2){
```

```
            # calculate the CFI up to that moment
            # I let the CFI grow also for the infected hosts, because they are
infected.
            # Of course they will not be considered in the calculation of the
next infection time, because they are already infected
            CFI <<- CFI +
beta*apply(matrix(hazardmatrix[which(indexI==1),],nrow=length(which(indexI==1)),t
otpoints),MARGIN=2,FUN=sum)*(time_event-tt)
            index_new_event <<- index_new_event + 1
            CFI_matrix[index_new_event,] <<- CFI
            # track the CFI over time
            timevector <<- rbind(timevector,time_event)  # track the time
            # update the status vector and the indices vectors for S and for I
            Current[,3] <<- id_
            Status[id_] <<- 3   #culled
            indexI[id_] <<- 3   #culled, it will not contribute to the infectious
matrix anymore
            indexS[id_] <<- 3   #culled, it is not susceptible anymore
            # save the number of infected over time in a list
            infected_over_time <<-
rbind(infected_over_time,c(time_event,length(indexI[indexI==1])))
            if(length(which(indexI==1))!=0){ # if there are still individual
infected
              # update the slope of the force of infection
              bb[which(indexS==0)] <<-
beta*apply(matrix(hazardmatrix[which(indexI==1),which(indexS==0)],nrow=length(whi
ch(indexI==1)), ncol=length(which(indexS==0))),MARGIN=2,FUN=sum)
              # calculate for each susceptible the next possible infection event
              t_infection[which(indexS==0)] <<- (Q_init[which(indexS==0)]-
CFI[which(indexS==0)])/bb[which(indexS==0)]
              t_infection[which(indexS==1|indexS==3)]  <<- 10000000
              # update the time
              tt <<- time_event
              next_infection_host <<- which.min(t_infection)
              next_infection_time <<- t_infection[which.min(t_infection)]
              # if the next infection host is already in the queue, you need to
remove the old one and add the new one with the     updated time of infection
              if ((next_infection_host%in%List_to_infect$id_host)==TRUE){
                List_to_infect <<- List_to_infect[-
(which((List_to_infect$id_host%in%next_infection_host)==TRUE)),]
              }
              List_to_infect <<-
rbind(List_to_infect,data.frame(Event_time=tt+next_infection_time,Type_event=rep(
2,length(next_infection_time)),id_host=next_infection_host))
              List_to_infect <<- List_to_infect[order(List_to_infect[,1]),]
            } else if (length(which(indexI==1)) == 0){# if there are no infection
events anymore the cumulative force infection of the susceptible should be set to
0
              #update the slope of the force of infection
              bb[which(indexS==0)] <<- 0
              List_to_infect <<- {}
            }
            return(1)}
}

### Start the simulation
# I first call the R file where I initialize the variables ("InitSim.R")
# Then I call the file ("SimLoop.R") that calls in a loop the function Event()
defined above

########## start the simulation #####################################
```

```
#ptm <- proc.time()
K<-100  # define the first infected
T_inf <- T_inf_matrix[K,]   # rgamma(totpoints,10, scale=7/10) # mean=7, std=2
Q_init <- Q_init_matrix[K,] # rexp(totpoints, rate = 1) # these are the
thresholds (exposure to infection) picked from an exponential distribution of
mean 1
source("InitSim.R") # initialization and setting of the first infected
while(nrow(Queue)!=0){
  source("Simloop.R")
}
# Show the simulation outputs
# The outputs are stored in the matrix History
head(History)
tail(History)

# code of InitSim.R sourced in the main code
# initialize vector status with 1 (all susceptibles)
Status <- matrix(1,nrow=totpoints)      # status is a vector recording the state
of each host
colnames(Status) <- "status"
#1= susceptible, 2=infectious, 3 culled
Queue <- {}
History <- {}
Current <- {}
infected_over_time <- {}
time_vector <- {}  # Initialize the Cumulative Force of Infection at the
begininng of the epidemic
CFI <- matrix(0,nrow=totpoints)
CFI_matrix <- matrix(0,ncol=totpoints,nrow=10000)
index_new_event <- 0
counter <- 0
# create vectors for the infected and the susceptibles, to use to compute the
cumulative force of infection
# These are index used for the calculation of the cumulative force of infection.
# They are used to keep track which hosts are infected or susceptible.
List_to_remove <- {}
List_to_infect <- {}
indexI <- matrix(0,nrow=totpoints)   #indexI==0, not yet infected, indexI==1
infected, indexI==3 culled
indexS <- matrix(0,nrow=totpoints)   # indexS==0, not yet infected, indexS==1
infected, indexS=3 culled
t_infection <- matrix(0,nrow=totpoints)
# coefficient of increase
bb <- matrix(0,nrow=totpoints)
tt <- 0 # start at time 0
next_infection_time <- 0
next_infection_host <- 0
#######  # initialize with the first one to be infected
firstone <- K
Status[firstone] <- 2 # now Status= 2 (infected)
indexI[firstone] <- 1 # infected
indexS[firstone] <- 1 # not susceptible anymore
#calculate coefficient of increase
bb[which(indexS==0)] <-
beta*apply(matrix(hazardmatrix[which(indexI==1),which(indexS==0)],nrow=length(whi
ch(indexI==1)),ncol=length(which(indexS==0))), MARGIN=2,FUN=sum) # In the rows
(i) the infected. in the j the supectibles
t_infection[which(indexS==0)] <- (Q_init[which(indexS==0)]-
CFI[which(indexS==0)])/bb[which(indexS==0)]
t_infection[which(indexS==1)] <- 10000000   # I set an extremely high number,
because it cannot infect itself
```

```
next_infection_time <- min(t_infection)
next_infection_host <- which.min(t_infection)
# add this infected host to the history vector
History <-
rbind(History,data.frame(Event_time=tt,Type_event=2,host_id=firstone,x_coord=Re(C
oord[as.numeric(firstone)]),y_coord=Im(Coord[as.numeric(firstone)]))) # record it
in the history vector. In the history vector add the coord
# update the list_to_infect and the list_to remove
List_to_infect <- rbind(List_to_infect,data.frame(Event_time=tt
+next_infection_time,Type_event=2,id_host = next_infection_host))
List_to_infect <- List_to_infect[order(List_to_infect[,1]),]
List_to_remove <- rbind(List_to_remove,data.frame(Event_time=tt
+T_inf[firstone],Type_event=3,id_host=firstone))
List_to_remove <- List_to_remove[order(List_to_remove[,1]),]
next_events <- rbind(List_to_infect[1,],List_to_remove[1,])
index_next_event <- which.min(next_events[,1])
Queue <- rbind(Queue,next_events[index_next_event,])
# now remove this event from the list_to_infect or list_to_remove
if(all.equal(cbind(List_to_infect[1,1],List_to_infect[1,2],List_to_infect[1,3]),c
bind(Queue[1,1],Queue[1,2],Queue[1,3]))==TRUE){
  List_to_infect <- List_to_infect[-c(1),]
} else
if(all.equal(cbind(List_to_remove[1,1],List_to_remove[1,2],List_to_remove[1,3]),c
bind(Queue[1,1],Queue[1,2],Queue[1,3]))==TRUE){
  List_to_remove <- List_to_remove[-c(1),]
}
# I add the current CFI (which is 0 at the beginning of the CFI_matrix)
index_new_event <- index_new_event+1
CFI_matrix[index_new_event,] <- CFI
timevector <- rbind(time_vector,tt)
infected_over_time <- rbind(infected_over_time,c(0,length(indexI[indexI==1])))

# code of SimLoop.R sourced in the main code
########### main loop between-herd model############################
Current <- Queue[1,]
Queue <- Queue[-(1),] # remove this event from the Queue
# update the status of that host
# call the function Event. If the function event returns 1, then save it in the
history vector
status_id <- Status[Current[,3]]
if (eval(event(Current[,1],Current[,2],status_id,Current[,3]))==1){  # If the
function event returns a new event save it in the history, and draw it on tyhe
map
   History <-
rbind(History,data.frame(Event_time=Current[,1],Type_event=Current[,2],host_id=Cu
rrent[,3],x_coord=Re(Coord[as.numeric(Current[,3])]),y_coord=Im(Coord[as.numeric(
Current[,3])]))) # record it in the history vector. In the history vector add the
coord
}
# to decide which is the next event compare the time of the 2 vectors (infected,
removed)
next_events <- rbind(List_to_infect[1,],List_to_remove[1,])
index_next_event <- which.min(next_events[,1])
Queue <- rbind(Queue,next_events[index_next_event,])
if(all.equal(cbind(List_to_infect[1,2],List_to_infect[1,3]),cbind(Queue[1,2],Queu
e[1,3]))==TRUE){
  List_to_infect <- List_to_infect[-c(1),]
} else
if(all.equal(cbind(List_to_remove[1,2],List_to_remove[1,3]),cbind(Queue[1,2],Queu
e[1,3]))==TRUE){
List_to_remove <- List_to_remove[-c(1),]
```

```
}
#####################################################################
```

Simulation data and comparison of simulation with approximation

Simulation data with four clusters

Distances between clusters based on centers of gravity of clusters

```
In[81]:= distance = {{0, 97.8631780678727, 156.182654236229, 126.383383714482},
    {97.8631780678727, 0, 166.54935834812, 83.0115444186513},
    {156.182654236229, 166.54935834812, 0, 94.4550453288847},
    {126.383383714482, 83.0115444186513, 94.4550453288847, 0}};
```

Individual reproduction numbers exceeding 1 with cluster identifier

```
In[82]:= allreproductionnumbers =
   {{1.148475296`, 1}, {1.120464798`, 1}, {1.522726886`, 1}, {1.638904507`, 1},
    {1.056185061`, 1}, {2.44241528`, 1}, {2.443808962`, 1}, {2.492162025`, 1},
    {2.907945375`, 1}, {2.838444857`, 1}, {2.772389752`, 1}, {2.712652444`, 1},
    {2.507462338`, 1}, {2.377117998`, 1}, {2.505137748`, 1}, {1.807290334`, 1},
    {1.423250259`, 1}, {1.14329519`, 1}, {1.988834769`, 1}, {2.346540781`, 1},
    {2.260801849`, 1}, {2.205322327`, 1}, {3.285707701`, 1}, {3.475002812`, 1},
    {3.141307853`, 1}, {3.1506694`, 1}, {3.189421497`, 1}, {3.895342081`, 1},
    {4.140160414`, 1}, {3.489943372`, 1}, {3.562001406`, 1}, {4.250697169`, 1},
    {4.216416548`, 1}, {3.504806282`, 1}, {3.9112991`, 1}, {3.444158271`, 1},
    {3.347788922`, 1}, {3.158224723`, 1}, {3.822718317`, 1}, {3.280525906`, 1},
    {3.151116626`, 1}, {3.475660801`, 1}, {3.245772409`, 1}, {3.039142992`, 1},
    {3.142585722`, 1}, {2.815570554`, 1}, {3.052002575`, 1}, {2.014566012`, 1},
    {2.233732856`, 1}, {2.685354367`, 1}, {2.539074016`, 1}, {3.863691658`, 1},
    {3.68378216`, 1}, {3.903681218`, 1}, {3.12402575`, 1}, {3.258467865`, 1},
    {3.950598552`, 1}, {3.29102214`, 1}, {3.117939939`, 1}, {4.273452975`, 1},
    {4.485321777`, 1}, {3.987719979`, 1}, {4.370151265`, 1}, {4.678565696`, 1},
    {4.385703665`, 1}, {4.346944513`, 1}, {4.125074415`, 1}, {4.724557338`, 1},
    {4.303096241`, 1}, {4.45096422`, 1}, {4.077746181`, 1}, {4.61923544`, 1},
    {4.3417651`, 1}, {4.274670025`, 1}, {4.880911712`, 1}, {5.275005408`, 1},
    {4.824743037`, 1}, {4.345004492`, 1}, {4.50677462`, 1}, {4.533433335`, 1},
    {4.648585487`, 1}, {5.010991555`, 1}, {4.832692696`, 1}, {4.856383343`, 1},
    {4.603263494`, 1}, {4.852096962`, 1}, {4.811114276`, 1}, {4.681814613`, 1},
    {5.075582751`, 1}, {4.753743853`, 1}, {5.297598487`, 1}, {4.614752492`, 1},
    {4.548312277`, 1}, {4.485068063`, 1}, {4.817116776`, 1}, {4.59012822`, 1},
    {3.86506976`, 1}, {4.60888731`, 1}, {4.050534236`, 1}, {4.787263759`, 1},
    {4.797980365`, 1}, {4.522976621`, 1}, {3.832121676`, 1}, {3.691757691`, 1},
    {3.388468987`, 1}, {2.934942262`, 1}, {2.919351423`, 1}, {2.835681837`, 1},
    {2.703931723`, 1}, {2.6155659`, 1}, {2.463504865`, 1}, {2.361426324`, 1},
    {2.123850412`, 1}, {2.268287519`, 1}, {1.863434934`, 1}, {1.724326601`, 1},
    {1.195191741`, 1}, {2.073832613`, 1}, {3.30427141`, 1}, {2.592692627`, 1},
    {3.539596266`, 1}, {4.042107508`, 1}, {3.989137789`, 1}, {4.129627024`, 1},
    {4.765106014`, 1}, {5.027767099`, 1}, {5.09228736`, 1}, {4.988084245`, 1},
    {5.423457751`, 1}, {5.281246303`, 1}, {5.310193954`, 1}, {5.181540657`, 1},
    {5.428256688`, 1}, {5.24868807`, 1}, {5.217269413`, 1}, {5.592804182`, 1},
    {5.456027392`, 1}, {5.296847798`, 1}, {5.180355293`, 1}, {5.087809884`, 1},
    {5.198860842`, 1}, {5.372058447`, 1}, {5.398154065`, 1}, {5.330431801`, 1},
    {5.382621156`, 1}, {5.346181728`, 1}, {5.192637216`, 1}, {5.295658377`, 1},
```

```
{5.439252614`, 1}, {5.416601725`, 1}, {5.207186866`, 1}, {5.367247923`, 1},
{5.503620769`, 1}, {5.269791431`, 1}, {5.240616385`, 1}, {5.220597042`, 1},
{5.433941382`, 1}, {5.445937619`, 1}, {5.342931697`, 1}, {5.123461184`, 1},
{4.572621537`, 1}, {4.853587667`, 1}, {4.618197203`, 1}, {4.735224445`, 1},
{4.518470743`, 1}, {4.768794897`, 1}, {4.65034822`, 1}, {4.908071612`, 1},
{4.17293612`, 1}, {3.886603863`, 1}, {4.023980579`, 1}, {4.329659894`, 1},
{4.099724391`, 1}, {3.671680112`, 1}, {3.226566377`, 1}, {2.555194905`, 1},
{2.709291564`, 1}, {2.33147429`, 1}, {1.750760617`, 1}, {1.991156799`, 1},
{1.423784173`, 1}, {1.64033569`, 1}, {2.223611784`, 1}, {2.340836508`, 1},
{2.276831894`, 1}, {2.396850032`, 1}, {2.660796193`, 1}, {2.912427465`, 1},
{3.451867789`, 1}, {3.395020473`, 1}, {4.981022199`, 1}, {5.068021308`, 1},
{4.576318056`, 1}, {4.269264281`, 1}, {4.557130424`, 1}, {5.41575424`, 1},
{5.498824683`, 1}, {5.293091324`, 1}, {5.218262447`, 1}, {5.529979076`, 1},
{5.582192501`, 1}, {5.110660104`, 1}, {5.57807335`, 1}, {5.348925489`, 1},
{5.46405602`, 1}, {5.406533369`, 1}, {4.939056362`, 1}, {5.37193633`, 1},
{5.323827456`, 1}, {5.31685416`, 1}, {5.322076308`, 1}, {5.495767768`, 1},
{5.259972769`, 1}, {5.51563431`, 1}, {5.34443105`, 1}, {5.492341358`, 1},
{5.383770921`, 1}, {5.46601835`, 1}, {5.567308507`, 1}, {5.515539148`, 1},
{5.547383652`, 1}, {5.550758209`, 1}, {5.440899112`, 1}, {5.418796876`, 1},
{5.368565836`, 1}, {5.315210381`, 1}, {4.702385626`, 1}, {4.835045223`, 1},
{4.587639231`, 1}, {4.882683869`, 1}, {4.588043043`, 1}, {4.831613678`, 1},
{4.549055091`, 1}, {4.965079718`, 1}, {4.92776524`, 1}, {5.155109399`, 1},
{5.342660061`, 1}, {5.36948434`, 1}, {4.435501539`, 1}, {5.076182138`, 1},
{5.174301143`, 1}, {4.717269425`, 1}, {3.714814589`, 1}, {3.71723458`, 1},
{4.048737095`, 1}, {3.167278598`, 1}, {2.896971114`, 1}, {2.867010089`, 1},
{2.059816878`, 1}, {2.238039764`, 1}, {1.934787847`, 1}, {1.166914899`, 1},
{2.398792457`, 1}, {2.022089407`, 1}, {1.866240547`, 1}, {2.756596112`, 1},
{2.626919952`, 1}, {3.459715034`, 1}, {3.602204211`, 1}, {3.191876827`, 1},
{3.248619019`, 1}, {3.100543762`, 1}, {3.353316293`, 1}, {4.387570266`, 1},
{3.864942244`, 1}, {4.250776394`, 1}, {4.29912852`, 1}, {4.055637745`, 1},
{3.984274311`, 1}, {4.484854854`, 1}, {4.708079239`, 1}, {4.501260382`, 1},
{4.983310871`, 1}, {4.963862236`, 1}, {4.871707679`, 1}, {4.282747085`, 1},
{4.465826467`, 1}, {5.299979366`, 1}, {5.028001567`, 1}, {4.583241737`, 1},
{4.616765076`, 1}, {5.081283771`, 1}, {4.608812891`, 1}, {4.862759674`, 1},
{4.691317355`, 1}, {5.146847669`, 1}, {4.961306691`, 1}, {4.631492453`, 1},
{4.973404297`, 1}, {4.624254688`, 1}, {4.890258974`, 1}, {4.99354838`, 1},
{4.899053024`, 1}, {4.937917288`, 1}, {4.751152071`, 1}, {4.152595871`, 1},
{4.077188268`, 1}, {4.293208841`, 1}, {4.87950771`, 1}, {4.402757919`, 1},
{4.055122787`, 1}, {4.237662699`, 1}, {3.300014916`, 1}, {3.751914498`, 1},
{4.263537725`, 1}, {3.398497297`, 1}, {3.362773006`, 1}, {2.224518599`, 1},
{1.73413814`, 1}, {2.263221822`, 1}, {2.13155161`, 1}, {2.309321919`, 1},
{3.421047765`, 1}, {3.131525823`, 1}, {3.633530168`, 1}, {3.50051632`, 1},
{4.129822602`, 1}, {4.058770755`, 1}, {4.171415196`, 1}, {4.127093596`, 1},
{4.175737561`, 1}, {4.054901131`, 1}, {3.872554107`, 1}, {3.60690557`, 1},
{3.796270056`, 1}, {2.787411408`, 1}, {2.963782198`, 1}, {2.643214557`, 1},
{2.263348264`, 1}, {2.642623663`, 1}, {1.325500106`, 1}, {1.902577312`, 1},
{2.268232254`, 1}, {2.899504023`, 1}, {2.579484499`, 1}, {2.957308578`, 1},
{2.406455499`, 1}, {1.447108243`, 1}, {1.948791571`, 1}, {2.00218429`, 1},
{2.378816115`, 1}, {2.292738965`, 1}, {2.278414707`, 1}, {1.131267984`, 1},
{1.380591373`, 1}, {1.76338844`, 1}, {1.807097969`, 1}, {1.721192805`, 1},
{1.521172303`, 1}, {1.112255792`, 1}, {1.038456266`, 1}, {1.11428319`, 1},
{1.106332335`, 1}, {1.229087618`, 1}, {1.230838428`, 1}, {1.210941154`, 1},
{1.370347712`, 1}, {1.012811581`, 1}, {1.020275325`, 2}, {1.000025225`, 2},
{1.225753395`, 2}, {1.476046005`, 2}, {1.769619733`, 2}, {1.600592855`, 2},
{2.02872208`, 2}, {1.655370357`, 2}, {1.783255203`, 2}, {1.954333696`, 2},
```

{1.785819366`, 2}, {2.838340721`, 2}, {2.623911363`, 2}, {2.816654399`, 2},
{2.556450178`, 2}, {2.683705917`, 2}, {2.523480832`, 2}, {2.956597959`, 2},
{1.428987397`, 2}, {1.635152466`, 2}, {1.57401872`, 2}, {1.462850689`, 2},
{2.086944511`, 2}, {1.813820295`, 2}, {2.298443345`, 2}, {2.393622204`, 2},
{2.553098303`, 2}, {2.226651238`, 2}, {3.434650353`, 2}, {3.008652504`, 2},
{3.313497361`, 2}, {3.183884841`, 2}, {2.839957239`, 2}, {3.308312284`, 2},
{3.528980865`, 2}, {3.116910372`, 2}, {3.442977656`, 2}, {3.640180426`, 2},
{3.375562704`, 2}, {3.505870744`, 2}, {3.251639885`, 2}, {3.278204402`, 2},
{3.662586272`, 2}, {3.307315118`, 2}, {3.288211451`, 2}, {3.433458585`, 2},
{3.420918`, 2}, {3.132063487`, 2}, {2.826639046`, 2}, {2.746479859`, 2},
{2.664786649`, 2}, {2.119171438`, 2}, {2.201547161`, 2}, {1.61266501`, 2},
{1.22669024`, 2}, {1.655073679`, 2}, {1.726478633`, 2}, {2.393303471`, 2},
{2.477175689`, 2}, {2.070335506`, 2}, {2.635601414`, 2}, {3.116546485`, 2},
{3.107792281`, 2}, {2.660266717`, 2}, {3.751051743`, 2}, {3.694486526`, 2},
{3.76696585`, 2}, {3.347219449`, 2}, {3.415536634`, 2}, {3.673803423`, 2},
{3.495599832`, 2}, {3.644917149`, 2}, {3.501534188`, 2}, {3.795398929`, 2},
{3.864305151`, 2}, {3.691462705`, 2}, {3.702212492`, 2}, {3.781252036`, 2},
{3.847833632`, 2}, {3.789278297`, 2}, {3.754773953`, 2}, {3.75517351`, 2},
{3.664799206`, 2}, {3.712548266`, 2}, {3.7751052`, 2}, {3.74487043`, 2},
{3.820843985`, 2}, {3.680516052`, 2}, {3.499049206`, 2}, {3.698347814`, 2},
{3.539091323`, 2}, {3.630816426`, 2}, {3.52164599`, 2}, {3.614622676`, 2},
{3.507447749`, 2}, {3.436102447`, 2}, {3.625188625`, 2}, {3.782360029`, 2},
{3.12750443`, 2}, {2.895494081`, 2}, {3.21500388`, 2}, {3.087406804`, 2},
{2.91939781`, 2}, {2.75490153`, 2}, {2.367909405`, 2}, {2.577353461`, 2},
{2.521756808`, 2}, {2.185693496`, 2}, {1.760274377`, 2}, {1.729825933`, 2},
{1.331704227`, 2}, {1.246410441`, 2}, {1.152122721`, 2}, {1.629110062`, 2},
{2.342573701`, 2}, {2.222576999`, 2}, {3.173229842`, 2}, {3.235026037`, 2},
{3.107386642`, 2}, {2.941274177`, 2}, {2.937968705`, 2}, {2.922814097`, 2},
{3.432783043`, 2}, {3.488808492`, 2}, {3.498539095`, 2}, {3.583286715`, 2},
{3.508913136`, 2}, {3.442507732`, 2}, {3.678194213`, 2}, {3.765810666`, 2},
{3.773044306`, 2}, {3.763381283`, 2}, {3.697075428`, 2}, {3.625900772`, 2},
{3.782082134`, 2}, {3.866073361`, 2}, {3.809168627`, 2}, {3.642266422`, 2},
{3.803949459`, 2}, {3.550078859`, 2}, {3.460987571`, 2}, {3.835932841`, 2},
{3.681199252`, 2}, {3.539094344`, 2}, {3.384234842`, 2}, {3.198348485`, 2},
{3.143480112`, 2}, {3.090227876`, 2}, {3.099474815`, 2}, {2.753526834`, 2},
{2.661058864`, 2}, {2.855217463`, 2}, {2.735753963`, 2}, {2.572106122`, 2},
{3.03363822`, 2}, {2.347027693`, 2}, {2.06425977`, 2}, {1.384091672`, 2},
{1.74996958`, 2}, {2.252841177`, 2}, {2.689593072`, 2}, {3.124902924`, 2},
{2.699202261`, 2}, {3.018243154`, 2}, {2.814084617`, 2}, {2.635946294`, 2},
{2.869892546`, 2}, {3.017902173`, 2}, {3.123908853`, 2}, {2.930886118`, 2},
{3.332682603`, 2}, {3.34385339`, 2}, {3.318358193`, 2}, {3.181691117`, 2},
{3.340858655`, 2}, {3.312217094`, 2}, {3.415597019`, 2}, {3.430599058`, 2},
{3.515465116`, 2}, {3.029988156`, 2}, {2.941218933`, 2}, {3.108424447`, 2},
{2.900721414`, 2}, {3.113122054`, 2}, {2.752200998`, 2}, {2.726412158`, 2},
{2.655870978`, 2}, {2.485970141`, 2}, {2.076269131`, 2}, {2.327363954`, 2},
{1.719690919`, 2}, {1.374777324`, 2}, {1.790040226`, 2}, {1.795281125`, 2},
{2.164362669`, 2}, {2.221541997`, 2}, {2.637504178`, 2}, {2.485724475`, 2},
{2.774713509`, 2}, {2.841479658`, 2}, {2.834126465`, 2}, {2.720176661`, 2},
{2.84268047`, 2}, {2.829010705`, 2}, {2.926657987`, 2}, {3.014715806`, 2},
{2.419942246`, 2}, {2.23328176`, 2}, {2.190732987`, 2}, {2.118969602`, 2},
{2.227493488`, 2}, {1.970833083`, 2}, {1.963265516`, 2}, {1.856140168`, 2},
{1.36032967`, 2}, {1.224709745`, 2}, {1.09870743`, 2}, {1.459355118`, 2},
{1.324843167`, 2}, {1.675747964`, 2}, {1.968305268`, 2}, {2.149363417`, 2},
{2.292359408`, 2}, {2.676881285`, 2}, {2.52819233`, 2}, {2.698809358`, 2},
{2.30999998`, 2}, {2.504876801`, 2}, {2.452379403`, 2}, {2.538780443`, 2},

{2.403581315`, 2}, {2.423527785`, 2}, {2.358564602`, 2}, {2.252773493`, 2},
{1.873660289`, 2}, {1.160667783`, 2}, {1.174648388`, 2}, {1.381177703`, 2},
{1.787310464`, 2}, {1.774608402`, 2}, {1.776284937`, 2}, {2.218970914`, 2},
{2.247783791`, 2}, {2.194960617`, 2}, {2.160843145`, 2}, {1.96991777`, 2},
{1.723778964`, 2}, {1.60312412`, 2}, {1.229671522`, 2}, {1.237123918`, 2},
{1.095163357`, 2}, {1.012684415`, 2}, {1.630722622`, 2}, {1.684321781`, 2},
{1.752387228`, 2}, {1.920017014`, 2}, {1.876973792`, 2}, {1.79393334`, 2},
{1.85668136`, 2}, {1.855538178`, 2}, {1.765007045`, 2}, {1.654249212`, 2},
{1.681387949`, 2}, {1.390483811`, 2}, {1.600651986`, 2}, {1.231330977`, 2},
{1.348856949`, 2}, {1.010425986`, 2}, {1.055432159`, 2}, {1.080034413`, 2},
{1.28134492`, 2}, {1.477888746`, 2}, {1.418885531`, 2}, {1.574700376`, 2},
{1.524647543`, 2}, {1.533032459`, 2}, {1.505213281`, 2}, {1.625520298`, 2},
{1.666473787`, 2}, {1.275270573`, 2}, {1.331581922`, 2}, {1.382136492`, 2},
{1.373992945`, 2}, {1.124025725`, 3}, {1.093445351`, 3}, {1.037912743`, 3},
{1.222216889`, 3}, {1.063192354`, 3}, {1.187272584`, 3}, {1.10388095`, 3},
{1.076690111`, 3}, {1.089989326`, 3}, {1.196980132`, 3}, {1.374074978`, 3},
{1.319706868`, 3}, {1.371997681`, 3}, {1.329341442`, 3}, {1.211398755`, 3},
{1.306737707`, 3}, {1.10205887`, 3}, {1.431893957`, 3}, {1.489198125`, 3},
{1.401846727`, 3}, {1.461069468`, 3}, {1.567929137`, 3}, {1.541627943`, 3},
{1.547644817`, 3}, {1.54101865`, 3}, {1.394112859`, 3}, {1.408206804`, 3},
{1.475712704`, 3}, {1.279122767`, 3}, {1.217973764`, 3}, {1.166316811`, 3},
{1.216397413`, 3}, {1.377112278`, 3}, {1.483729736`, 3}, {1.425864399`, 3},
{1.491459304`, 3}, {1.564346976`, 3}, {1.629340403`, 3}, {1.623673389`, 3},
{1.608090829`, 3}, {1.616467188`, 3}, {1.539524024`, 3}, {1.591800281`, 3},
{1.387721251`, 3}, {1.26429608`, 3}, {1.166434827`, 3}, {1.270713946`, 3},
{1.545714573`, 3}, {1.653704519`, 3}, {1.676466955`, 3}, {1.630311006`, 3},
{1.656204217`, 3}, {1.550524467`, 3}, {1.592789718`, 3}, {1.266911587`, 3},
{1.502409006`, 3}, {1.698979868`, 3}, {1.769830556`, 3}, {1.908256745`, 3},
{1.892469362`, 3}, {1.751273232`, 3}, {1.862465672`, 3}, {1.616735643`, 3},
{1.585290293`, 3}, {1.150746048`, 3}, {1.045383832`, 3}, {1.308564713`, 3},
{1.351688129`, 3}, {1.528846214`, 3}, {1.754035725`, 3}, {1.643616876`, 3},
{1.637512405`, 3}, {2.023876368`, 3}, {1.792142924`, 3}, {2.01322981`, 3},
{1.999239548`, 3}, {2.039773491`, 3}, {2.086400768`, 3}, {2.040539038`, 3},
{1.946381443`, 3}, {1.934972625`, 3}, {2.041931619`, 3}, {1.864455056`, 3},
{1.644229809`, 3}, {1.47234625`, 3}, {1.170357207`, 3}, {1.049286098`, 3},
{1.073768138`, 3}, {1.405766463`, 3}, {1.325125649`, 3}, {1.631139261`, 3},
{1.65109098`, 3}, {1.899323411`, 3}, {2.091018386`, 3}, {2.037738515`, 3},
{2.156299627`, 3}, {2.187224898`, 3}, {2.16842198`, 3}, {2.106147039`, 3},
{2.229347625`, 3}, {2.224753846`, 3}, {2.19879621`, 3}, {2.205852414`, 3},
{2.151575771`, 3}, {2.05431408`, 3}, {2.128958286`, 3}, {1.110912549`, 3},
{1.144369124`, 3}, {1.29960862`, 3}, {1.588183634`, 3}, {1.931584664`, 3},
{1.952880597`, 3}, {2.205621489`, 3}, {2.142327079`, 3}, {2.319391499`, 3},
{1.92288077`, 3}, {2.080516766`, 3}, {1.647056149`, 3}, {1.102812632`, 3},
{1.062801582`, 3}, {1.162266014`, 3}, {1.264896664`, 3}, {1.488447834`, 3},
{1.44159884`, 3}, {2.006143909`, 3}, {1.885609464`, 3}, {2.29447285`, 3},
{2.312077946`, 3}, {2.175050621`, 3}, {2.609844157`, 3}, {2.573809454`, 3},
{2.555055945`, 3}, {2.433491511`, 3}, {2.615387521`, 3}, {2.370531768`, 3},
{2.490624012`, 3}, {2.559390221`, 3}, {2.096633209`, 3}, {2.32155685`, 3},
{1.427878743`, 3}, {1.283783113`, 3}, {1.206639571`, 3}, {2.138464709`, 3},
{2.177564123`, 3}, {2.368386872`, 3}, {2.552317596`, 3}, {2.624240999`, 3},
{2.495177487`, 3}, {2.613061231`, 3}, {2.628369334`, 3}, {2.559696252`, 3},
{2.546368417`, 3}, {2.654005875`, 3}, {2.597565951`, 3}, {2.637260269`, 3},
{2.650778411`, 3}, {2.483391018`, 3}, {2.647475474`, 3}, {2.649637191`, 3},
{2.561817071`, 3}, {2.488462548`, 3}, {2.167904234`, 3}, {2.439541061`, 3},
{2.398430657`, 3}, {2.178187063`, 3}, {1.899156269`, 3}, {1.279450165`, 3},

{1.622148142`, 3}, {1.600103325`, 3}, {2.014027105`, 3}, {2.107291744`, 3},
{2.10560101`, 3}, {2.612446336`, 3}, {2.459225371`, 3}, {2.582952862`, 3},
{2.472351433`, 3}, {2.604566038`, 3}, {2.500370043`, 3}, {2.620844477`, 3},
{2.494026819`, 3}, {2.553589023`, 3}, {2.480785665`, 3}, {2.026050862`, 3},
{2.156384164`, 3}, {1.423751874`, 3}, {1.382879821`, 3}, {1.701621702`, 3},
{2.123020495`, 3}, {2.171330272`, 3}, {2.288616929`, 3}, {2.340118886`, 3},
{2.268793439`, 3}, {2.403934976`, 3}, {2.382835581`, 3}, {2.222074145`, 3},
{2.023268617`, 3}, {2.219060309`, 3}, {2.171932544`, 3}, {2.027614231`, 3},
{1.950270251`, 3}, {1.999799235`, 3}, {2.224644235`, 3}, {2.225456993`, 3},
{1.938496236`, 3}, {2.071171472`, 3}, {1.160787906`, 3}, {1.485232864`, 3},
{2.177763571`, 3}, {2.173763625`, 3}, {2.247239803`, 3}, {2.272097573`, 3},
{2.255535112`, 3}, {2.008048807`, 3}, {1.653931522`, 3}, {1.823840177`, 3},
{1.460364785`, 3}, {1.41064471`, 3}, {2.238012494`, 3}, {2.367492606`, 3},
{2.288350976`, 3}, {2.313329572`, 3}, {2.208697302`, 3}, {2.105201059`, 3},
{2.329185106`, 3}, {2.313505918`, 3}, {2.313180311`, 3}, {2.357001115`, 3},
{2.323032754`, 3}, {2.340608178`, 3}, {2.281832934`, 3}, {2.078168916`, 3},
{1.74266609`, 3}, {1.881917619`, 3}, {1.810519998`, 3}, {2.314209994`, 3},
{2.325528557`, 3}, {2.217866432`, 3}, {2.406430335`, 3}, {2.388614728`, 3},
{2.346935351`, 3}, {2.360608942`, 3}, {2.405010105`, 3}, {2.409549354`, 3},
{2.051328653`, 3}, {2.380766931`, 3}, {2.35838458`, 3}, {2.305043893`, 3},
{2.30596249`, 3}, {2.40298475`, 3}, {2.404793284`, 3}, {2.390699087`, 3},
{2.419801119`, 3}, {2.40358978`, 3}, {2.295747124`, 3}, {2.327386977`, 3},
{2.163152139`, 3}, {1.970541938`, 3}, {1.175339003`, 3}, {1.171107781`, 3},
{1.133661287`, 3}, {1.922175752`, 3}, {1.782204379`, 3}, {2.047035543`, 3},
{2.094236639`, 3}, {2.383229933`, 3}, {2.277260595`, 3}, {2.431985049`, 3},
{2.383686789`, 3}, {2.213596874`, 3}, {1.909266812`, 3}, {1.685689004`, 3},
{1.805295018`, 3}, {1.151095239`, 3}, {1.763371788`, 3}, {1.951470756`, 3},
{2.148623231`, 3}, {2.647831403`, 3}, {2.56121272`, 3}, {2.777788011`, 3},
{2.559183476`, 3}, {1.155030806`, 3}, {1.035159811`, 3}, {1.228074446`, 3},
{2.087414338`, 3}, {2.016949332`, 3}, {2.502970085`, 3}, {2.407610644`, 3},
{2.495542154`, 3}, {2.455773918`, 3}, {2.197588286`, 3}, {2.343819946`, 3},
{2.470887038`, 3}, {2.690798472`, 3}, {2.575407086`, 3}, {2.614256376`, 3},
{2.946775556`, 3}, {2.894691118`, 3}, {2.783535595`, 3}, {2.915275915`, 3},
{2.928734224`, 3}, {2.986641093`, 3}, {3.002591602`, 3}, {3.022625479`, 3},
{3.060349035`, 3}, {2.814606586`, 3}, {2.705633228`, 3}, {2.609882223`, 3},
{2.364349539`, 3}, {2.651649458`, 3}, {2.476764734`, 3}, {2.04228539`, 3},
{1.871789055`, 3}, {1.571123788`, 3}, {1.588632557`, 3}, {1.216724834`, 3},
{1.226875287`, 3}, {1.241955819`, 3}, {1.688101601`, 3}, {2.500201574`, 3},
{2.293311561`, 3}, {2.797463315`, 3}, {2.583962019`, 3}, {2.793366758`, 3},
{2.730659634`, 3}, {2.601886755`, 3}, {3.002057273`, 3}, {3.024612957`, 3},
{2.863950408`, 3}, {3.041657481`, 3}, {3.068994907`, 3}, {3.072225431`, 3},
{3.02942364`, 3}, {3.047462383`, 3}, {3.075205944`, 3}, {3.144462286`, 3},
{3.138603785`, 3}, {3.139080439`, 3}, {3.109028921`, 3}, {3.139651189`, 3},
{3.086453978`, 3}, {3.134571469`, 3}, {3.099075059`, 3}, {2.956891426`, 3},
{2.902716188`, 3}, {2.95877177`, 3}, {3.000104311`, 3}, {3.017225554`, 3},
{2.944568914`, 3}, {2.791098181`, 3}, {2.865763937`, 3}, {2.882367167`, 3},
{3.031013817`, 3}, {2.417281459`, 3}, {2.186775091`, 3}, {1.842983864`, 3},
{2.003714999`, 3}, {1.070186878`, 3}, {1.288347094`, 3}, {1.813541834`, 3},
{1.994167023`, 3}, {2.072335137`, 3}, {2.607769661`, 3}, {2.512653609`, 3},
{2.467368723`, 3}, {2.580308648`, 3}, {2.358605993`, 3}, {3.061192606`, 3},
{2.801101094`, 3}, {2.620754617`, 3}, {2.994136666`, 3}, {2.891630837`, 3},
{2.743197084`, 3}, {2.954607787`, 3}, {2.70879344`, 3}, {2.984878266`, 3},
{2.874594853`, 3}, {2.697468032`, 3}, {3.035426527`, 3}, {2.842056439`, 3},
{2.607748369`, 3}, {2.677924116`, 3}, {2.877487851`, 3}, {2.705679319`, 3},
{2.852091417`, 3}, {2.580983916`, 3}, {2.717885239`, 3}, {2.616204295`, 3},

{2.628103083`, 3}, {2.501368601`, 3}, {1.960932774`, 3}, {2.379859029`, 3},
{1.78449731`, 3}, {1.919739508`, 3}, {2.0647789`, 3}, {2.030132248`, 3},
{1.870480647`, 3}, {2.357594875`, 3}, {2.04252891`, 3}, {2.145379825`, 3},
{2.16875969`, 3}, {2.662051466`, 3}, {2.45533119`, 3}, {2.118275162`, 3},
{2.478064708`, 3}, {2.214269207`, 3}, {1.963560577`, 3}, {1.248743085`, 3},
{1.018212988`, 3}, {1.413325568`, 3}, {1.391355548`, 3}, {1.351724639`, 3},
{1.916038307`, 3}, {1.880251288`, 3}, {1.369499308`, 3}, {1.106398236`, 3},
{1.076999716`, 3}, {1.195681477`, 3}, {1.144564054`, 3}, {1.16904513`, 4},
{1.31352463`, 4}, {1.005027452`, 4}, {1.038385575`, 4}, {1.012765913`, 4},
{1.437175599`, 4}, {1.369799453`, 4}, {1.462068857`, 4}, {1.546735296`, 4},
{1.769603582`, 4}, {1.754781246`, 4}, {1.360393243`, 4}, {1.516318363`, 4},
{1.257091242`, 4}, {1.052190297`, 4}, {1.362524856`, 4}, {1.526164877`, 4},
{1.663872454`, 4}, {1.458276717`, 4}, {1.672393224`, 4}, {1.605652495`, 4},
{1.618606337`, 4}, {1.971780339`, 4}, {1.775558631`, 4}, {1.778972043`, 4},
{1.819709433`, 4}, {2.107808244`, 4}, {2.181218436`, 4}, {2.022245334`, 4},
{2.167737533`, 4}, {1.957704193`, 4}, {2.145282263`, 4}, {2.217325604`, 4},
{2.316061472`, 4}, {2.002931518`, 4}, {2.153936533`, 4}, {2.225893605`, 4},
{2.022773977`, 4}, {1.853374847`, 4}, {2.025543111`, 4}, {1.886210246`, 4},
{1.63696224`, 4}, {1.73805766`, 4}, {1.557504271`, 4}, {1.410990659`, 4},
{1.377940969`, 4}, {1.766936523`, 4}, {1.790619037`, 4}, {2.193449206`, 4},
{2.171836717`, 4}, {2.167660943`, 4}, {2.255950848`, 4}, {2.216884979`, 4},
{2.388885739`, 4}, {2.317354484`, 4}, {2.490741093`, 4}, {2.392071331`, 4},
{2.437497669`, 4}, {2.429492734`, 4}, {2.338423044`, 4}, {2.548125065`, 4},
{2.425941942`, 4}, {2.313780875`, 4}, {2.460776099`, 4}, {2.446495896`, 4},
{2.141471336`, 4}, {2.173854647`, 4}, {2.209578332`, 4}, {2.043503902`, 4},
{2.001733059`, 4}, {1.720226936`, 4}, {2.062173485`, 4}, {1.655755373`, 4},
{1.28990484`, 4}, {1.335569778`, 4}, {1.15284707`, 4}, {1.372199138`, 4},
{1.383985537`, 4}, {1.526628262`, 4}, {1.752440438`, 4}, {2.025853392`, 4},
{1.916606958`, 4}, {1.771098762`, 4}, {1.770504648`, 4}, {2.20707691`, 4},
{2.298648889`, 4}, {2.159595465`, 4}, {2.119269566`, 4}, {2.091755234`, 4},
{2.50781341`, 4}, {2.509563368`, 4}, {2.480940401`, 4}, {2.487059503`, 4},
{2.513784003`, 4}, {2.50632038`, 4}, {2.447682694`, 4}, {2.54037227`, 4},
{2.519311424`, 4}, {2.555496977`, 4}, {2.551562879`, 4}, {2.547468234`, 4},
{2.4800542`, 4}, {2.48822977`, 4}, {2.451299578`, 4}, {2.412689397`, 4},
{2.422474542`, 4}, {2.402790748`, 4}, {2.50461547`, 4}, {2.291637876`, 4},
{2.42569337`, 4}, {2.350012963`, 4}, {2.093229876`, 4}, {2.260909074`, 4},
{2.214431995`, 4}, {2.347710834`, 4}, {2.11264915`, 4}, {1.612053109`, 4},
{1.577312287`, 4}, {1.605181042`, 4}, {1.630279591`, 4}, {1.134343099`, 4},
{1.073097388`, 4}, {1.055083033`, 4}, {1.080816054`, 4}, {1.432030007`, 4},
{2.106985598`, 4}, {2.045083349`, 4}, {2.269227718`, 4}, {2.111311105`, 4},
{2.135693822`, 4}, {2.228094019`, 4}, {2.402101487`, 4}, {2.227687558`, 4},
{2.334562351`, 4}, {2.114935751`, 4}, {2.342867814`, 4}, {1.989963475`, 4},
{1.761539156`, 4}, {1.785375477`, 4}, {1.66939901`, 4}, {1.587271503`, 4},
{1.315999777`, 4}, {1.226931577`, 4}, {1.403554493`, 4}, {1.150679029`, 4},
{1.156612307`, 4}, {1.273086889`, 4}, {1.208096206`, 4}, {1.333336081`, 4},
{1.189608662`, 4}, {1.258157466`, 4}, {1.212698712`, 4}, {1.270782282`, 4},
{1.163586322`, 4}, {1.653982363`, 4}, {1.830995444`, 4}, {1.631042563`, 4},
{1.97981533`, 4}, {1.729699549`, 4}, {1.813113931`, 4}, {1.970475258`, 4},
{1.708479815`, 4}, {2.050337175`, 4}, {2.06848875`, 4}, {1.812996653`, 4},
{1.618802249`, 4}, {1.171107413`, 4}, {1.067088106`, 4}, {1.239139561`, 4},
{1.269536853`, 4}, {1.382479442`, 4}, {1.253073899`, 4}, {1.463398265`, 4},
{1.429288145`, 4}, {1.386923898`, 4}, {1.419624657`, 4}, {1.385189944`, 4},
{1.451259071`, 4}, {1.485545915`, 4}, {1.252530367`, 4}, {1.127548885`, 4},
{1.300622224`, 4}, {1.582597113`, 4}, {1.351492574`, 4}, {1.408545899`, 4},
{1.42936667`, 4}, {1.198033576`, 4}, {1.028844807`, 4}, {1.038750845`, 4},

```
{1.206835051`, 4}, {1.344668313`, 4}, {1.383400022`, 4}, {1.492873544`, 4},
{1.495685273`, 4}, {1.409879604`, 4}, {1.411230782`, 4}, {1.428311594`, 4},
{1.357395142`, 4}, {1.369067098`, 4}, {1.334361278`, 4}, {1.018436459`, 4},
{1.356993508`, 4}, {1.325256286`, 4}, {1.435159563`, 4}, {1.314597822`, 4},
{1.351392911`, 4}, {1.338976214`, 4}, {1.453320468`, 4}, {1.435424066`, 4},
{1.371085738`, 4}, {1.391511185`, 4}, {1.22599791`, 4}, {1.178928347`, 4},
{1.160408893`, 4}, {1.088450614`, 4}, {1.045740933`, 4}, {1.002650927`, 4}};
```

Reproduction numbers per cluster

In[83]:=
```
Print["number of clusters : ", nclusters = Length[distance], "\n"];
```

```
number of clusters : 4
```

In[84]:=
```
Do[cluster[i] = Select[allreproductionnumbers, #[[2]] == i &][[All, 1]],
  {i, 1, nclusters}];
Do[Print["mean, variance, shape and scale of individual reproduction
     numbers for cluster ", i , " : ", mean[i] = Mean[cluster[i]], "\t",
   var[i] = Variance[cluster[i]], "\t", shape[i] = mean[i]^2/var[i],
   "\t", scale[i] = var[i]/mean[i]], {i, 1, nclusters}];
reproductionnumbers = Table[mean[i], {i, 1, nclusters}];
```

```
mean, variance, shape and scale of individual reproduction numbers for cluster
 1 : 3.82292    1.6639    8.78341    0.435244

mean, variance, shape and scale of individual reproduction numbers for cluster
 2 : 2.57817    0.698602    9.51467    0.270968

mean, variance, shape and scale of individual reproduction numbers for cluster
 3 : 2.08025    0.324342    13.3422    0.155915

mean, variance, shape and scale of individual reproduction numbers for cluster
 4 : 1.75429    0.218452    14.0879    0.124524
```

In[87]:=
```
Print["number of hosts per cluster : ",
  n = Table[Length[cluster[i]], {i, nclusters}], "\n"];
```

```
number of hosts per cluster : {358, 283, 418, 217}
```

Plot of reproduction numbers

In[88]:=
```
Do[Print[
  Show[Plot[PDF[GammaDistribution[shape[i], scale[i]], x], {x, 0.5, 7}, Filling → Axis],
   Histogram[{cluster[i]}, 10, "PDF"], PlotRange → All]], {i, 1, nclusters}]
```

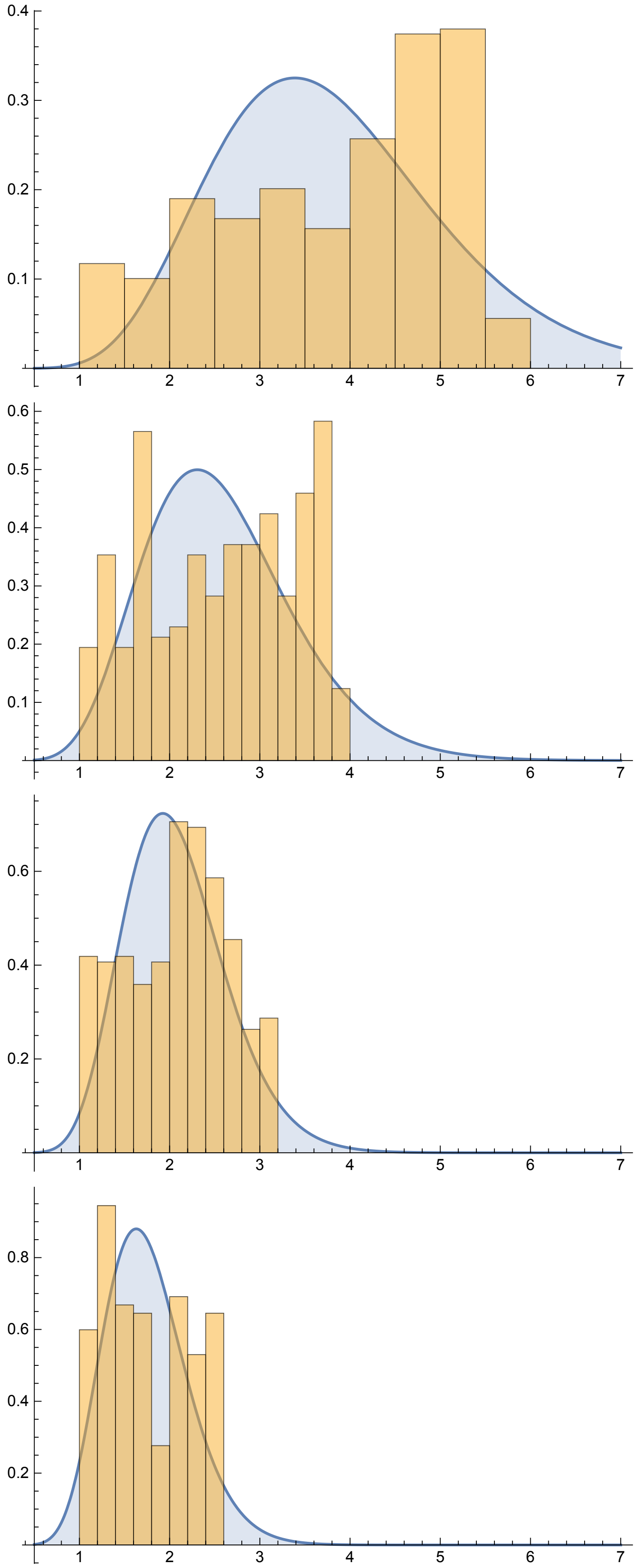

Probabilities of a large outbreak

Probability generating function of Z

```
In[89]:= g[s_, i_ ] := (1 + (mean[i] / shape[i]) (1 - s)) ^ (-shape[i])

In[90]:= Do[pl[i] = FindRoot[g[1 - q, i] == 1 - q , {q, 0.6}][[1, 2]], {i, 1, nclusters}];

In[91]:= Do[Print["cluster ", i, " : probability of a large outbreak : ", pl[i]],
          {i, 1, nclusters}]

cluster 1 : probability of a large outbreak : 0.952461

cluster 2 : probability of a large outbreak : 0.865201

cluster 3 : probability of a large outbreak : 0.786216

cluster 4 : probability of a large outbreak : 0.683707
```

Final size in clusters

Standard final size using means of individual reproduction numbers

```
In[92]:= Do[zstandard[i] = NSolve[z == 1 - Exp[-reproductionnumbers[[i]] z], z][[2, 1, 2]],
           {i, 1, nclusters}]  // Quiet
```

Final size based on individual reproduction numbers (and assumption that hosts are independent and topology unimportant). See Miller JC (2012) Bull Math Biol 74, 2125-2141

```
In[93]:= Do[zmiller[i] =
           FindRoot[z == 1 - (1 / Length[cluster[i]]) Sum[Exp[- z cluster[i][[j]]], {j,
                  1, Length[cluster[i]]}], {z, 0.6}][[1, 2]], {i, 1, nclusters}]
```

Final size based on assumption of gamma distribution of reproduction numbers (and independence assumption). See Katriel (2012) J Math Biol 65, 237-262

```
In[94]:= Do[zkatriel[i] =
           FindRoot[z == 1 - (1 + (mean[i] z) / shape[i]) ^- (shape[i]), {z, 0.6}][[1, 2]], {i,
           1, nclusters}]
```

Print calculations

```
In[95]:= Do[Print["cluster ", i, " : standard final size (fraction): ",
           zstandard[i], "\tfinal size (Miller) : ", zmiller[i],
           "\tfinal size (Katriel): ", zkatriel[i]], {i, 1, nclusters}]

cluster 1 : standard final size (fraction): 0.976039
     final size (Miller) : 0.940922     final size (Katriel): 0.952461

cluster 2 : standard final size (fraction): 0.902356
     final size (Miller) : 0.859401     final size (Katriel): 0.865201

cluster 3 : standard final size (fraction): 0.817382
     final size (Miller) : 0.783579     final size (Katriel): 0.786216

cluster 4 : standard final size (fraction): 0.714458
     final size (Miller) : 0.683046     final size (Katriel): 0.683707
```

In[96]:=
```
Do[Print["cluster ", i, " : standard final size (numbers): ",
  zstandard[i] n[[i]], "\tfinal size (Miller) : ", zmiller[i] n[[i]],
  "\tfinal size (Katriel): ", zkatriel[i] n[[i]]], {i, 1, nclusters}]
```

```
cluster 1 : standard final size (numbers): 349.422
     final size (Miller) : 336.85    final size (Katriel): 340.981
cluster 2 : standard final size (numbers): 255.367
     final size (Miller) : 243.21    final size (Katriel): 244.852
cluster 3 : standard final size (numbers): 341.666
     final size (Miller) : 327.536    final size (Katriel): 328.638
cluster 4 : standard final size (numbers): 155.037
     final size (Miller) : 148.221    final size (Katriel): 148.364
```

Spatial transmission kernel

In[97]:=
```
h[x_] := h0 / (1 + (x / r0) ^α);
rule = {h0 → 0.005, r0 → 1.9, α → 2.1, T → 7, c → 10};
```

Cluster to cluster transmission

Probability of a large outbreak in i given an introduction in j

In[99]:=
```
p[i_, j_] :=
 If[i == j, 0, 1 - Exp[-pl[i] n[[i]] (1 - Exp[- (1 - (c / (c + T h[distance[[i, j]]])) ^c)])
     zmiller[j] n[[j]] pl[j]]] /. rule
```

Probability of a large outbreak in i given an introduction in j, conditioned on large outbreak in j

In[100]:=
```
pc[i_, j_] :=
 If[i == j, 0, 1 - Exp[-pl[i] n[[i]] (1 - Exp[- (1 - (c / (c + T h[distance[[i, j]]])) ^c)])
     zmiller[j] n[[j]]]] /. rule
```

(Modelled) direct and one-step cluster-to-cluster infection probabilities - notice: for 4 clusters, can be generalised. number of routes from j to i while visiting every intermediate at most once can be very large. for instance, if all hosts are visited, the number of combinations is \prod_{k=0}^{n-1} n-2-k. if l hosts are visited, the number of combination is \prod_{k=0}^{l-1} n-2-k (with appropriate range for l)

In[101]:=
```
allclusters = Table[i, {i, nclusters}];
r[i_, j_] := Block[{comp = Complement[allclusters, {i, j}]},
  1 - (1 - p[i, j]) Product[(1 - pc[i, k] * p[k, j]), {k, comp}]
    (1 - pc[i, comp[[1]]] * pc[comp[[1]], comp[[2]]] * p[comp[[2]], j])
    (1 - pc[i, comp[[2]]] * pc[comp[[2]], comp[[1]]] * p[comp[[1]], j])]
```

Overall result

In[103]:=
```
Do[If[i ≠ j, Print["probability of large outbreak in ", i, " given introduction in ",
   j, " : r(", i, ", ", j, ") = ", r[i, j]]], {i, 1, nclusters}, {j, 1, nclusters}]
```

```
probability of large outbreak in 1 given introduction in 2 : r(1, 2) = 0.563281
probability of large outbreak in 1 given introduction in 3 : r(1, 3) = 0.412684
probability of large outbreak in 1 given introduction in 4 : r(1, 4) = 0.372733
probability of large outbreak in 2 given introduction in 1 : r(2, 1) = 0.595186
probability of large outbreak in 2 given introduction in 3 : r(2, 3) = 0.395299
probability of large outbreak in 2 given introduction in 4 : r(2, 4) = 0.402723
probability of large outbreak in 3 given introduction in 1 : r(3, 1) = 0.469786
probability of large outbreak in 3 given introduction in 2 : r(3, 2) = 0.424235
probability of large outbreak in 3 given introduction in 4 : r(3, 4) = 0.387547
probability of large outbreak in 4 given introduction in 1 : r(4, 1) = 0.466901
probability of large outbreak in 4 given introduction in 2 : r(4, 2) = 0.475569
probability of large outbreak in 4 given introduction in 3 : r(4, 3) = 0.427852
```

Comparison with simulations

Observed probabilities of large outbreak in i given introduction in j (with operational threshold of 10 infected hosts)

In[104]:=
```
q[i_, j_] := Which[i == 1 && j == 2, 0.551,
  i == 1 && j == 3, 0.44,
  i == 1 && j == 4, 0.359,
  i == 2 && j == 1, 0.601,
  i == 2 && j == 3, 0.395,
  i == 2 && j == 4, 0.35,
  i == 3 && j == 1, 0.556,
  i == 3 && j == 2, 0.491,
  i == 3 && j == 4, 0.387,
  i == 4 && j == 1, 0.539,
  i == 4 && j == 2, 0.544,
  i == 4 && j == 3, 0.447]
```

Absolute error (approximation - simulation) and relative error

In[105]:=
```
abserror[i_, j_] := Evaluate[If[i == j, 0, (r[i, j] - q[i, j])]]
Print["mean absolute error : ",
 Sum[Abs[abserror[i, j]], {i, 1, nclusters}, {j, 1, nclusters}] /
  (nclusters * (nclusters - 1))]
relerror[i_, j_] := Evaluate[If[i == j, 0, (r[i, j] - q[i, j]) / q[i, j]]]
Print["mean relative error : ",
 Sum[Abs[relerror[i, j]], {i, 1, nclusters}, {j, 1, nclusters}] /
  (nclusters * (nclusters - 1))]
```

```
mean absolute error : 0.0354474
mean relative error : 0.0732112
```

Simulation and approximation for avian influenza in poultry in the Netherlands

Poultry farms in high-density areas in the Netherlands

```
In[109]:= distance = {{0, 88.62}, {88.62, 0}};

In[110]:= allreproductionnumbers = {{2.519411724`, 1}, {1.675181836`, 1},
    {1.674327066`, 1}, {1.591636301`, 1}, {1.506983895`, 1}, {2.103285498`, 1},
    {1.253559036`, 1}, {1.808613619`, 1}, {1.481922273`, 1}, {1.754203231`, 1},
    {1.840495073`, 1}, {1.71648653`, 1}, {1.442648714`, 1}, {1.7727936`, 1},
    {2.421988834`, 1}, {1.851934033`, 1}, {2.014545951`, 1}, {2.016369511`, 1},
    {1.878728439`, 1}, {1.331577808`, 1}, {1.127154927`, 1}, {1.889064475`, 1},
    {1.10832657`, 1}, {1.130090969`, 1}, {1.135872145`, 1}, {2.513951694`, 1},
    {1.152233232`, 1}, {2.292004411`, 1}, {1.754905083`, 1}, {2.426070523`, 1},
    {2.294835701`, 1}, {1.227115273`, 1}, {1.271006612`, 1}, {2.115576647`, 1},
    {2.289731681`, 1}, {1.56088241`, 1}, {1.773125927`, 1}, {1.488160538`, 1},
    {2.471904049`, 1}, {1.846155388`, 1}, {1.896504511`, 1}, {1.457424252`, 1},
    {1.034373883`, 1}, {1.032441243`, 1}, {1.367251413`, 1}, {1.215054825`, 1},
    {1.090543274`, 1}, {1.001257086`, 1}, {2.205100765`, 1}, {1.70345806`, 1},
    {1.540820911`, 1}, {1.442600465`, 1}, {1.59808322`, 1}, {1.881590328`, 1},
    {2.104295593`, 1}, {1.016221006`, 1}, {1.821122108`, 1}, {1.307592545`, 1},
    {1.338955842`, 1}, {1.944157555`, 1}, {1.032819487`, 1}, {1.621657008`, 1},
    {1.519776531`, 1}, {1.082059271`, 1}, {1.982845234`, 1}, {1.074664155`, 1},
    {1.590420474`, 1}, {1.114941276`, 1}, {2.113556158`, 1}, {1.629205755`, 1},
    {1.050110517`, 1}, {2.058425754`, 1}, {2.468162092`, 1}, {2.379806973`, 1},
    {1.657674379`, 1}, {1.315435048`, 1}, {1.414996745`, 1}, {2.149427677`, 1},
    {1.574324239`, 1}, {1.77480352`, 1}, {2.420875677`, 1}, {2.463819696`, 1},
    {1.438949981`, 1}, {1.44597268`, 1}, {1.201348742`, 1}, {1.054389102`, 1},
    {2.066336389`, 1}, {1.056213798`, 1}, {1.030095341`, 1}, {1.650892658`, 1},
    {2.0297726`, 1}, {1.694016687`, 1}, {2.418375787`, 1}, {1.595920521`, 1},
    {1.5279701`, 1}, {2.111373315`, 1}, {2.457543105`, 1}, {1.39964175`, 1},
    {1.118917522`, 1}, {2.425847104`, 1}, {1.302873469`, 1}, {1.63594634`, 1},
    {1.667845129`, 1}, {1.686330256`, 1}, {1.154978276`, 1}, {1.315073759`, 1},
    {1.800927829`, 1}, {1.481049702`, 1}, {1.608691871`, 1}, {2.098587719`, 1},
    {2.212845492`, 1}, {2.023451965`, 1}, {2.041453689`, 1}, {1.956054853`, 1},
    {1.615458471`, 1}, {1.170536245`, 1}, {1.722338992`, 1}, {1.341020017`, 1},
    {1.671987381`, 1}, {1.643594199`, 1}, {2.108805597`, 1}, {1.552265949`, 1},
    {1.412948702`, 1}, {1.415680548`, 1}, {1.630061947`, 1}, {1.424058124`, 1},
    {1.201949999`, 1}, {1.122529585`, 1}, {2.13136292`, 1}, {1.997598243`, 1},
    {2.378093129`, 1}, {1.912555735`, 1}, {1.529320323`, 1}, {1.553976223`, 1},
    {1.940452068`, 1}, {1.216665838`, 1}, {1.933888034`, 1}, {1.331398729`, 1},
    {2.110163313`, 1}, {1.47957507`, 1}, {1.422394795`, 1}, {1.756793549`, 1},
    {1.209509173`, 1}, {1.891584698`, 1}, {2.344668901`, 1}, {2.461349945`, 1},
    {2.065211348`, 1}, {1.807683602`, 1}, {2.122656066`, 1}, {1.510503007`, 1},
    {1.111065279`, 1}, {1.110455289`, 1}, {1.028332647`, 1}, {1.062931005`, 1},
    {2.07402237`, 1}, {1.63450002`, 1}, {2.295210397`, 1}, {2.460849346`, 1},
    {2.132579441`, 1}, {2.004168765`, 1}, {2.011134596`, 1}, {1.936805203`, 1},
    {2.115199528`, 1}, {1.578727638`, 1}, {1.945325533`, 1}, {2.446566711`, 1},
```

```
{1.570158358`, 1}, {1.497815854`, 1}, {1.746089775`, 1}, {1.810521638`, 1},
{1.609976529`, 1}, {1.073062625`, 1}, {2.03149558`, 1}, {1.080679089`, 1},
{2.253542109`, 1}, {2.362989635`, 1}, {2.00844807`, 1}, {1.845729601`, 1},
{1.838298491`, 1}, {1.234313025`, 1}, {1.61597124`, 1}, {1.637498023`, 1},
{1.349706752`, 1}, {2.188797105`, 1}, {2.026311827`, 1}, {1.979468858`, 1},
{1.40652308`, 1}, {2.005838828`, 1}, {1.838850671`, 1}, {1.02792565`, 1},
{2.225469109`, 1}, {1.924328001`, 1}, {1.521674719`, 1}, {2.338917919`, 1},
{1.771395435`, 1}, {1.187360012`, 1}, {1.94843266`, 1}, {1.742458363`, 1},
{1.555052103`, 1}, {1.496209086`, 1}, {1.508131754`, 1}, {2.093528015`, 1},
{1.53467055`, 1}, {2.485479865`, 1}, {2.321825688`, 1}, {1.113576404`, 1},
{1.936607962`, 1}, {1.665630599`, 1}, {1.940486495`, 1}, {1.508465666`, 1},
{2.533518462`, 1}, {1.893231894`, 1}, {2.094618868`, 1}, {1.571357294`, 1},
{1.639594924`, 1}, {2.123277818`, 1}, {1.252345813`, 1}, {1.114448188`, 1},
{1.085554393`, 1}, {1.548877213`, 1}, {2.472090038`, 1}, {1.92577405`, 1},
{1.690295745`, 1}, {1.683474574`, 1}, {1.99101201`, 1}, {2.18177123`, 1},
{1.448683582`, 1}, {2.264608725`, 1}, {1.912865358`, 1}, {2.040700423`, 1},
{1.567183186`, 1}, {1.00047102`, 1}, {2.01319415`, 1}, {2.268069001`, 1},
{2.00538552`, 1}, {2.005978832`, 1}, {1.10124041`, 1}, {1.560044968`, 1},
{1.730826341`, 1}, {1.149149602`, 1}, {1.07941427`, 1}, {1.815815141`, 1},
{1.049438899`, 1}, {1.117369095`, 1}, {2.445060169`, 1}, {1.143903116`, 1},
{1.953000308`, 1}, {1.644127682`, 1}, {2.381422406`, 1}, {2.018449413`, 1},
{1.464147268`, 1}, {1.4944527`, 1}, {1.934239937`, 1}, {1.784700364`, 1},
{2.122426336`, 1}, {1.538148311`, 1}, {2.156832614`, 1}, {1.848351975`, 1},
{1.013355743`, 1}, {2.08200221`, 1}, {2.517882506`, 1}, {1.945562769`, 1},
{2.344347547`, 1}, {2.34746734`, 1}, {1.927587196`, 1}, {2.095231716`, 1},
{1.201815382`, 1}, {1.00751079`, 1}, {2.175454905`, 1}, {1.411470926`, 1},
{1.045277735`, 1}, {1.665713338`, 1}, {1.588448082`, 1}, {1.237958921`, 1},
{2.093880044`, 1}, {2.268618756`, 1}, {1.571515341`, 1}, {2.28821729`, 1},
{2.242284289`, 1}, {1.770690148`, 1}, {1.837113523`, 1}, {2.315694644`, 1},
{1.937951787`, 1}, {1.229294207`, 1}, {1.49188953`, 1}, {1.526103544`, 1},
{1.637079463`, 1}, {1.833304842`, 1}, {1.036757881`, 1}, {1.57339272`, 1},
{2.042551648`, 1}, {1.372316764`, 1}, {1.604453369`, 1}, {1.451534675`, 1},
{1.589628043`, 1}, {1.4868462`, 1}, {1.053212053`, 1}, {1.002470902`, 1},
{1.727206207`, 1}, {2.059195014`, 1}, {1.809490666`, 1}, {1.330285388`, 1},
{1.43590445`, 1}, {1.248177815`, 2}, {1.113401346`, 2}, {1.183251029`, 2},
{1.22359807`, 2}, {1.127698836`, 2}, {1.471551387`, 2}, {1.208963189`, 2},
{1.370308937`, 2}, {1.273693001`, 2}, {1.249227174`, 2}, {1.475361278`, 2},
{1.570723741`, 2}, {1.203523453`, 2}, {1.519696876`, 2}, {1.552366387`, 2},
{1.255897619`, 2}, {1.11520251`, 2}, {1.315430732`, 2}, {1.488840339`, 2},
{1.458046676`, 2}, {1.154092522`, 2}, {1.088754238`, 2}, {1.19504768`, 2},
{1.111298572`, 2}, {1.063122447`, 2}, {1.394757139`, 2}, {1.541741457`, 2},
{1.326702197`, 2}, {1.076426839`, 2}, {1.554843899`, 2}, {1.221516995`, 2},
{1.127219207`, 2}, {1.204818903`, 2}, {1.389982264`, 2}, {1.063202454`, 2},
{1.159862933`, 2}, {1.332025877`, 2}, {1.479238536`, 2}, {1.393998119`, 2},
{1.202651116`, 2}, {1.070219852`, 2}, {1.040936169`, 2}, {1.061903149`, 2},
{1.05057091`, 2}, {1.03791262`, 2}, {1.1580123`, 2}, {1.123646293`, 2},
{1.505314649`, 2}, {1.067933781`, 2}, {1.048514755`, 2}, {1.131710493`, 2},
{1.38585948`, 2}, {1.19622639`, 2}, {1.036607467`, 2}, {1.562294194`, 2},
{1.042219655`, 2}, {1.164329828`, 2}, {1.251269703`, 2}, {1.467563142`, 2},
{1.128746631`, 2}, {1.185256673`, 2}, {1.099793878`, 2}, {1.094082012`, 2},
{1.44873016`, 2}, {1.092917605`, 2}, {1.237594335`, 2}, {1.239611188`, 2}};
```

Reproduction numbers in high-density areas

In[111]:=
```
Print["number of clusters : ", nclusters = Length[distance], "\n"];
```

```
number of clusters : 2
```

In[112]:=
```
Do[cluster[i] = Select[allreproductionnumbers, #[[2]] == i &][[All, 1]],
  {i, 1, nclusters}];
Do[Print["mean, variance, shape and scale of individual reproduction
     numbers for cluster ", i , " : ", mean[i] = Mean[cluster[i]], "\t",
   var[i] = Variance[cluster[i]], "\t", shape[i] = mean[i]^2 / var[i],
   "\t", scale[i] = var[i] / mean[i]], {i, 1, nclusters}];
reproductionnumbers = Table[mean[i], {i, 1, nclusters}];
```

```
mean, variance, shape and scale of individual reproduction numbers for cluster
 1 : 1.71207	0.174687	16.7797	0.102033

mean, variance, shape and scale of individual reproduction numbers for cluster
 2 : 1.24531	0.0273038	56.7983	0.0219252
```

In[115]:=
```
Print["number of hosts per cluster : ",
  n = Table[Length[cluster[i]], {i, nclusters}], "\n"];
```

```
number of hosts per cluster : {303, 67}
```

Plot of reproduction numbers

In[116]:=
```
Do[Print[Show[Plot[PDF[GammaDistribution[shape[i], scale[i]], x],
     {x, 0.5, 4}, Filling → Axis, PlotRange → All],
   Histogram[{cluster[i]}, 10, "PDF"], PlotRange → All]], {i, 1, nclusters}]
```

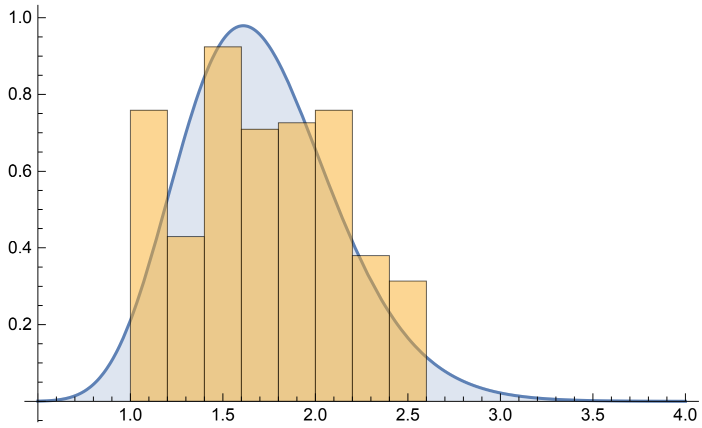

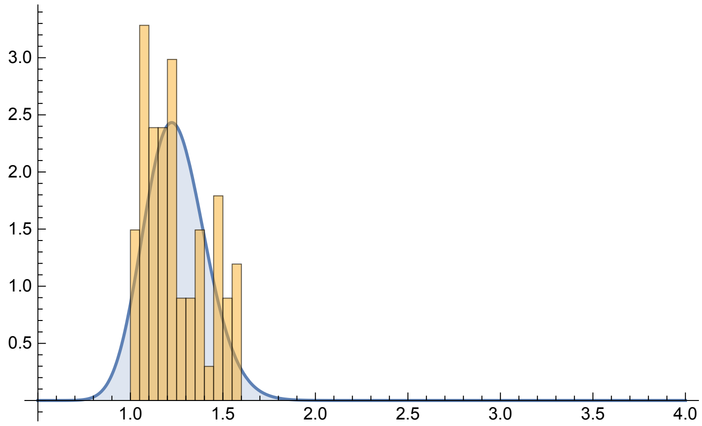

Probabilities of a large outbreak in high-density areas

In[117]:=
```
g[s_, i_] := (1 + (mean[i] / shape[i]) (1 - s)) ^ (-shape[i]) (* PGF of Z *)
Do[pl[i] = FindRoot[g[1 - q, i] == 1 - q, {q, 0.6}][[1, 2]], {i, 1, nclusters}];
Do[Print["cluster ", i, " : probability of a large outbreak : ", pl[i]],
 {i, 1, nclusters}]
```

```
cluster 1 : probability of a large outbreak : 0.670642

cluster 2 : probability of a large outbreak : 0.360811
```

Final size in high-density areas

Standard final size using means of individual reproduction numbers

In[120]:=
```
Do[zstandard[i] = NSolve[z == 1 - Exp[-reproductionnumbers[[i]] z], z][[2, 1, 2]],
  {i, 1, nclusters}] // Quiet
```

Final size based on individual reproduction numbers (and assumption that hosts are independent). See Miller JC (2012) Bull Math Biol 74, 2125-2141

In[121]:=
```
Do[zmiller[i] =
  FindRoot[z == 1 - (1 / Length[cluster[i]]) Sum[Exp[- z cluster[i][[j]]], {j,
        1, Length[cluster[i]]}], {z, 0.6}][[1, 2]], {i, 1, nclusters}]
```

Final size based on assumption of gamma distribution of reproduction numbers (and independence asumption). See Katriel (2012) J Math Biol 65, 237-262

In[122]:=
```
Do[zkatriel[i] =
  FindRoot[z == 1 - (1 + (mean[i] z) / shape[i]) ^- (shape[i]), {z, 0.6}][[1, 2]], {i,
  1, nclusters}]
```

Print calculations

In[123]:=
```
Do[Print["cluster ", i, " : standard final size : ",
  zstandard[i], "\tfinal size (Miller) : ", zmiller[i],
  "\tfinal size (Katriel): ", zkatriel[i]], {i, 1, nclusters}]
```

```
cluster 1 : standard final size : 0.696554
    final size (Miller) : 0.669838    final size (Katriel): 0.670642

cluster 2 : standard final size : 0.366237
    final size (Miller) : 0.360925    final size (Katriel): 0.360811
```

Spatial transmission kernel

In[124]:=
```
hh[x_] := h0 / (1 + (x / r0) ^α);
rule = {h0 → 0.008, r0 → 1.9, α → 2.1, T → 7, c → 10};
(* notice increased height *)
```

Transmission between high-density areas

Probability of a large outbreak in i given an introduction in j

In[126]:=
```
p[i_, j_] :=
 If[i == j, 0, 1 - Exp[-pl[i] n[[i]] (1 - Exp[- (1 - (c / (c + T h[distance[[i, j]]])) ^c)])
     zmiller[j] n[[j]] pl[j]]] /. rule
```

Probability of a large outbreak in i given an introduction in j, conditioned on large outbreak in j

In[127]:=
```
pc[i_, j_] :=
 If[i == j, 0, 1 - Exp[-pl[i] n[[i]] (1 - Exp[- (1 - (c / (c + T h[distance[[i, j]]])) ^c)])
     zmiller[j] n[[j]]]] /. rule
```

In[128]:=
```
allclusters = Table[i, {i, nclusters}];
r[i_, j_] :=
 1 - (1 - p[i, j]) Product[(1 - p[i, k] * p[k, j]), {k, Complement[allclusters, {i, j}]}]
```

Overall result

In[130]:=
```
Do[If[i ≠ j, Print["probability of large outbreak in i given introduction in j : r(",
   i, ", ", j, ") = ", r[i, j]]], {i, 1, nclusters}, {j, 1, nclusters}]
```

```
probability of large outbreak in i given introduction in j : r(1, 2) = 0.0305904

probability of large outbreak in i given introduction in j : r(2, 1) = 0.0560282
```

Comparison with simulations

Observed probabilities of large outbreak in i given introduction in j (with operational threshold of 10 infected hosts)

In[131]:=
```
q[i_, j_] := Which[i == 1 && j == 2, 0.075,
  i == 2 && j == 1, 0.076]
```

Absolute error (approximation - simulation) and relative error

In[132]:=
```
abserror[i_, j_] := Evaluate[If[i == j, 0, (r[i, j] - q[i, j])]]
Print["mean absolute error : ",
 Sum[Abs[abserror[i, j]], {i, 1, nclusters}, {j, 1, nclusters}] /
  (nclusters * (nclusters - 1))]
relerror[i_, j_] := Evaluate[If[i == j, 0, (r[i, j] - q[i, j]) / q[i, j]]]
Print["mean relative error : ",
 Sum[Abs[relerror[i, j]], {i, 1, nclusters}, {j, 1, nclusters}] /
  (nclusters * (nclusters - 1))]
```

```
mean absolute error : 0.0321907

mean relative error : 0.427457
```
